# Dual PTP1B/DPP4 Inhibitory Potential of *Agathosma betulina*, *Cymbopogon citratus*, and *Artemisia afra*: Structure-Based Modeling of Phytochemical Leads and Essential Oil Bioassays

**DOI:** 10.34133/csbj.0028

**Published:** 2026-04-16

**Authors:** Oluwaseye Adedirin, Saheed Sabiu

**Affiliations:** Department of Biotechnology and Food Science, Faculty of Applied Sciences, Durban University of Technology, Durban 4000, South Africa.

## Abstract

Type 2 diabetes mellitus (T2DM) remains a global health challenge characterized by insulin resistance and impaired incretin effects. This has spurred research into potential therapeutic agents from natural sources that can inhibit both protein tyrosine phosphatase 1B (PTP1B) and dipeptidyl peptidase 4 (DPP4), which are implicated in these diabetes effects. This study explored the therapeutic potential of *Artemisia afra*, *Cymbopogon citratus*, and *Agathosma betulina* through a dual approach: in vitro enzyme inhibition assays of their essential oils and computational modeling of their broader characteristic phytochemicals. Experimental results identified *A. betulina* essential oil as the most potent source, yielding IC_11_ values of 27.26 μg/ml for PTP1B and 42.28 μg/ml for DPP4. To explore the wider polypharmacological potential of these botanical sources beyond the volatile fraction, computational screening of the plant metabolites was done, which highlighted nonvolatile flavonoids quercetin-3,7-diglucoside (Q37DG) and quercetin-7-O-glucoside (Q7G) as primary theoretical leads of *A. betulina*. MMGBSA analysis confirmed exceptional binding affinities for Q37DG (Δ*G*_bind_ of −52.19 kcal/mol for PTP1B and −58.23 kcal/mol for DPP4), driven by van der Waals interactions and engagement with critical catalytic residues (Glu^117^, Asp^183^, Glu^206^, and Ser^630^). DFT calculations established that molecular softness and LUMO energies are key descriptors of inhibitory reactivity. While genetic algorithm QSAR models (*R*^2^ > 0.88) validated the theoretical potency of these leads, pharmacokinetic profiling identified important translation bottlenecks, including low gastrointestinal absorption. Consequently, these flavonoids are characterized as early-stage pharmacological probes and structural templates for multi-target optimization. This is interdisciplinary research that identifies botanical polypharmacological leads for diabetes.

## Graphical Abstract

**Figure:**
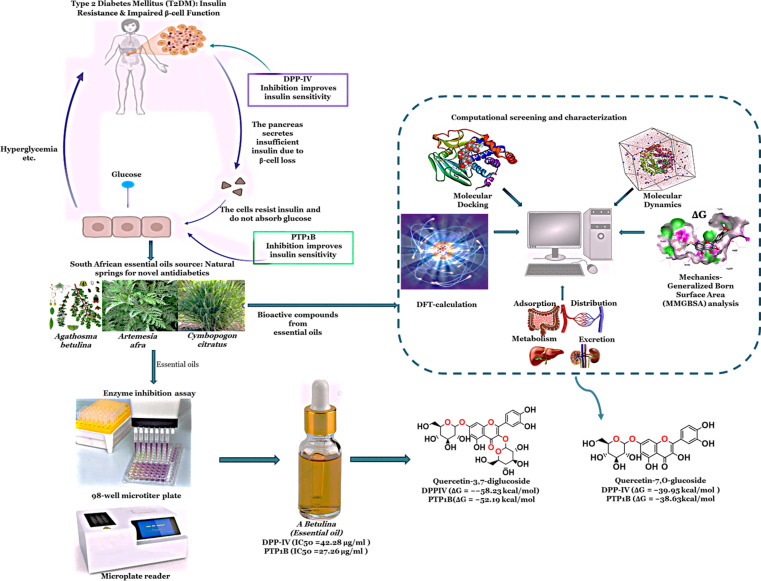

## Introduction

The global diabetes epidemic presents a formidable public health challenge, with South Africa facing a severe burden, with 4.2 million adults (12% of the population) affected by the disease [1]. The burden varies significantly by ethnicity, with Indian South Africans showing the highest prevalence, followed by White and Colored populations, while Black South Africans have the lowest rates. Diabetes ranks as the country’s second leading cause of death after tuberculosis [2]. The situation is compounded by poor healthcare detection, with 72.6% of cases going undiagnosed, highlighting critical gaps in screening and early intervention systems [3]. Modern type 2 diabetes mellitus (T2DM) treatment increasingly uses dual-target approaches to address the disease’s complex mechanisms. Protein tyrosine phosphatase 1B (PTP1B) and dipeptidyl peptidase 4 (DPP4) are promising therapeutic targets with complementary actions. PTP1B inhibition enhances insulin sensitivity and may reduce leptin resistance, providing metabolic benefits that include weight management. DPP4 inhibitors (“gliptins”) preserve incretin hormones, such as GLP-1, promoting glucose-dependent insulin release and glucagon suppression without causing hypoglycemia. This dual strategy targets both major T2DM defects: insulin resistance and β-cell dysfunction [4].

The exploration of indigenous South African medicinal plants as sources of novel antidiabetic compounds has gained considerable scientific interest, particularly regarding their essential oil constituents and bioactive extracts. *Agathosma betulina* (Buchu), for instance, contains metabolites with the potential to inhibit PTP1B, thereby enhancing insulin receptor sensitivity—a critical mechanism for managing T2DM [5]. Furthermore, aqueous extracts of Buchu have been shown to normalize glucose levels in diabetic rats and increase glucose uptake in 3T3-L1 cell lines [6,7]. Similarly, *Artemisia afra* is recognized as one of the most frequently used medicinal plants in South Africa for diabetes, with documented traditional usage in the Eastern Cape and proven efficacy in decreasing blood glucose levels in experimental models [8–11]. *Cymbopogon citratus* (Lemongrass) further complements this profile by modulating blood glucose levels, improving insulin sensitivity, and protecting pancreatic β-cell function through antioxidant and anti-inflammatory pathways [12]. Its essential oils and teas exhibit hypoglycemic and hypolipidemic potential while reducing post-prandial hyperglycemia through the inhibition of α-amylase and α-glucosidase enzymes [13–15].

While these studies have examined individual essential oils for their antidiabetic properties, a comprehensive computational screening of essential oil constituents for their potential as PTP1B/DPP4 inhibitors has not been fully explored. Structure-based modeling (such as molecular docking) directly calculates protein–ligand interactions, while quantitative structure–activity relationship (QSAR) (ligand-based modeling) can predict the biological activities of the ligand. Thus, combining ligand- and structure-based methods can improve predictability, leading to the identification and design of more effective lead compounds. These computational strategies enable rapid screening of multiple phytochemicals, elucidating binding mechanisms and interaction patterns with target proteins [8]. It is on this background that this study investigated the inhibitory potential of the essential oil constituents of *A. afra*, *C. citratus*, and *A. betulina* against PTP1B and DPP4 using molecular docking, molecular dynamics (MD), binding energy analysis, quantum mechanical methods, enzyme inhibition assay, and machine learning (ML)-driven QSAR study. This work aims to identify bioactive compounds with dual inhibitory potential against the enzymes and develop innovative therapeutic strategies for preventing and managing type 2 diabetes.

## Materials and Methods

### Compound selection and preparation

Metabolites representing the broader phytochemical profile of *A. betulina*, *C. citratus*, and *A. afra* were mined from J. A. Duke’s database [13] and published articles [6,16–18]. While the in vitro assays focused specifically on the volatile essential oil fraction, the computational study adopted an integrated in silico strategy. This involved data mining to identify major polar metabolites, such as flavonoid glycosides, to investigate the multi-target potential of the whole plant’s bioactive matrix and provide a detailed understanding of their antidiabetic properties through molecular docking and dynamics. Metabolites’ 3-dimensional (3D) molecular structures were obtained from PubChem (https://pubchem.ncbi.nlm.nih.gov/), and their molecular geometry was refined using the B3LYP/6-31G** density function theory method. Refined molecular structures in SDF format were imported into PyRx 8.0 (https://pyrx.sourceforge.io/) for conversion into PDBQT format for subsequent use [5].

### Target enzyme preparation

X-ray crystal structures of human PTP1B (PDB ID: 5t19, 2.10-Å resolution, residues His^0^-Asp^298^) and dipeptidyl peptidase 4 (DPP4; PDB ID: 5t4e, 1.17-Å resolution, residues Thr^39^-Pro^766^) were retrieved from the Protein Data Bank (https://www.rcsb.org/). Both structures contained co-crystallized ligands. Water molecules, redundant chains, and nonessential heteroatoms were removed using PyMOL software (http://pymol.org). Polar hydrogen atoms were subsequently added, and active sites were defined based on co-crystallized ligand positions. Kollman charges were assigned, Gasteiger charges were computed, and AD4 atom types were applied using AutoDock Tools in PyRx 8.0, with final conversion to PDBQT format, thereby optimizing them for molecular docking [19].

### Molecular docking protocol

Molecular docking was performed using AutoDock Vina (https://vina.scripps.edu/) implemented in PyRx 8.0, following the methodology of Trott and Olson [20]. Grid maps were generated around the identified binding sites with center coordinates of (−2.56, 64.01, 5.21) and dimensions of (21.43, 22.74, 15.82) Å for PTP1B, and center coordinates of (34.43, 50.88, 37.75) with dimensions of (25.00, 21.78, 25.00) Å for DPP4. Docking calculations employed an exhaustiveness parameter of 8 and utilized AutoDock Vina’s hybrid algorithm, combining global stochastic search with local gradient-based optimization [20]. The molecular docking protocol was optimized and validated through systematic grid map generation centered on the identified 3D coordinates of each enzyme binding site. The native co-crystallized ligand associated with each target was extracted and subjected to iterative redocking procedures within the designated binding site. Grid parameters were systematically adjusted to ensure that redocking simulations yielded a conformation approximating the ligand’s known crystallographic pose [5].

### MD simulation protocols

Docked molecules were ranked based on their docking score, and the top 5 with the highest absolute docking score were selected for each enzyme for MD simulation studies. The MD simulations were performed using AMBER 18 on the South African Center for High Performance Computing (CHPC) Lengau HPC HEAL1361 clusters (https://www.chpc.ac.za/), following the method of Case et al. [21] with modifications. The complexes of the top 5 compounds with PTP1B or DPP4 were separated into ligand and receptor components using UCSF Chimera. ANTECHAMBER assigned atom types to ligands using the General AMBER Force Field (GAFF) and AM1-BCC charge method, while enzymes were parameterized with the ff14SB force field via T-leap. The combined systems were solvated in TIP3P water boxes (12.0 Å buffer), and Cl^−^ and Na^+^ ions were added, as needed, to neutralize the charge. Following energy minimization, heating, and equilibration phases, 120-ns production MD simulations were conducted using SANDER with the leapfrog Verlet integrator and SHAKE algorithm at 2-fs time steps. Simulations employed NPT ensemble conditions (300 K via Langevin thermostat with 1.0 ps^−1^ collision frequency, 1 bar via barostat pressure coupling). Furthermore, Cpptraj (C++ rewritten process trajectory) was used to refine the trajectory file for quantitative analyses of post-dynamic parameters, which include the following: root mean square deviation (RMSD) of bound complexes from that of unbound enzyme α carbon atoms; root mean square fluctuation (RMSF); radius of gyration (ROG), which explains changes in enzymes compactness; and solvent-accessible surface area (SASA), which is indicative of conformational changes in protein structure and changes in the number of hydrogen bonds (nH-bond) in the enzyme because of binding with the compounds.

### Binding free energy calculations

Post-dynamics analysis was conducted using Cpptraj present in AMBER 18 for molecular mechanics-generalized born surface area (MMGBSA) free energy calculations, following established protocols with modifications [22]. Trajectory frames were sampled at 40-frame intervals across the entire 120,000 frames. The generalized Born (GB) solvation model was employed with physiological salt concentration (0.15 M). Per-residue energy decomposition analysis was performed across the entire sequences of the enzymes (PTP1B: residues 1 to 299; DPP4: residues 39 to 766). Binding free energies (Δ*G*_bind_) were calculated using the thermodynamic cycle: Δ*G*_bind_ = Δ*G*_complex_ − (Δ*G*_receptor_ + Δ*G*_ligand_). Δ*G*_complex_, Δ*G*_receptor_, and Δ*G*_ligand_ are the free energies of the bound complex, unbound receptor, and unbound ligand states, respectively. In this study, however, it should be noted that configurational entropy contributions (−TΔS) were not calculated due to the prohibitive computational cost and extensive memory requirements associated with their calculation via normal mode analysis (NMODE) and the size of these protein–ligand systems. Therefore, the reported values represent effective binding enthalpy and serve as relative indicators of binding affinity rather than absolute thermodynamic free energy.

### Quantum mechanical descriptors

Density functional theory (DFT) calculations were performed using the B3LYP/6-31G** method in Spartan 14 to optimize molecular geometries of the top 5 compounds. Frontier molecular orbital properties, including highest occupied molecular orbital (HOMO) and lowest unoccupied molecular orbital (LUMO) energies, were obtained from the optimized structures. Other quantum mechanical descriptors were calculated using established equations [23]: energy Δ*E* = *E*_LUMO_ – *E*_HOMO_, ionization potential (IP = −*E*_HOMO_), electron affinity (EA = −*E*_LUMO_), electronegativity [χ = (IP + EA)/2], hardness [η = (IP – EA)/2], softness (ξ = 1/2η), chemical potential (μ = −χ), and electrophilicity index (ω = μ^2^/2η) to characterize molecular reactivity profiles.

### Pharmacokinetic studies

Metabolic and toxicological profiles of selected top 5 compounds with inhibition potential against PTP1B and DPP4 were done using the SwissADME online tool (http://www.swissadme.ch). The method employed involves multiple steps of converting the chemical structure into simplified molecular input line entry system (SMILES) format and uploading the same into the SwissADME portal for the prediction of several pharmacokinetic features, such as gastrointestinal (GI) absorption, distribution, metabolism, and excretion (ADME) properties, blood–brain barrier permeability, cytochrome P450 inhibitors, P-glycoprotein substrates, bioavailability score, number of hydrogen bond acceptors and donors, Lipinski violation, lipophilicity, and synthetic accessibility [24].

### In vitro evaluation of PTP1B and DPP4 inhibition by the essential oils

The inhibitory activity of the *A. betulina*, *C. citratus*, and *A. afra* essential oils (Natural Organics, South Africa) against DPP4 (Sigma-Aldrich, Germany) was evaluated using the procedure reported Vijayakumar et al. with slight modifications [25]. A stock solution was prepared by dissolving 5 μl of the essential oil sample [which weighed 0.0089 g (*A. betulina*), 0.0109 g (*C. citratus*), and 0.0111g (*A. afra*)] in 1 ml of 10% dimethyl sulfoxide (DMSO) in assay buffer (50 mM tris–HCl, pH 7.5). Serial dilutions were performed from this stock to obtain working (test) solutions ranging from 10 to 150 μg/ml. Subsequently, 20 μl of each test solution was aliquoted into a 96-well microplate, followed by the addition of 140 μl of assay buffer. Twenty microliters of recombinant soluble human DPP4 enzyme, prepared in 50 ml of 5 mM tris–HCl assay buffer, was then added, and the reaction mixture was preincubated at 37 °C for 10 min. The enzymatic reaction was initiated by adding 20 μl of 1 mM H-Gly-Pro-pNA substrate (Sigma-Aldrich, Germany). The mixture was further incubated for 45 min at 37 °C, and absorbance was measured at 405 nm using a Thermo-Scientific Multiskan Go microplate reader (F1-01620, Vantaa, Finland). Sitagliptin (Sigma-Aldrich, Germany) served as a positive control, while the assay buffer was used as a negative control.

The in vitro inhibitory activities of the essential oil samples against PTP1B were assessed using the Calbiochem PTP1B colorimetric assay kit (User Protocol; 2008, catalog no.: 539736, USA), with ursolic acid serving as a reference compound [26]. Essential oil stock solutions and working (test) concentrations (10 to 200 μg/ml) were prepared as previously described. Thirty-five microliters of 1× assay buffer (prepared by diluting the 2× buffer provided in the kit 1:1 with distilled water) was added to a 96-well microplate. Subsequently, 10 μl of the essential oil solution and 5 μl of human recombinant PTP1B enzyme (5 μg/ml) were sequentially added to each well. The reaction was initiated by adding 50 μl of PTP1B substrate (IRS), pre-warmed to the assay temperature (30 °C). The reaction mixture was then incubated for 30 min at 30 °C. The reaction was terminated by adding 25 μl of red reagent, followed by an additional 20-min incubation for color development. Absorbance was measured at 620 nm using a Thermo Scientific Multiskan Go microplate reader (F1-01620, Vantaa, Finland) at the conclusion of the incubation period. Ursolic acid (Sigma-Aldrich) was utilized as a positive control, and 1× assay buffer was utilized as a negative control. Percentage inhibition at each concentration was calculated from 3 independent measurements using the following formula: % Inhibition = [(Absorbance_blank_ − Absorbance_test_)/Absorbance_blank_] × 100. Nonlinear regression analysis was performed using GraphPad Prism 8.0 software (GraphPad Software Inc., La Jolla, CA, USA) to determine the half-maximal inhibitory concentration (IC_50_) values.

### GA-QSAR model construction and lead compound IC_50_ value prediction

**Fig. 1.**
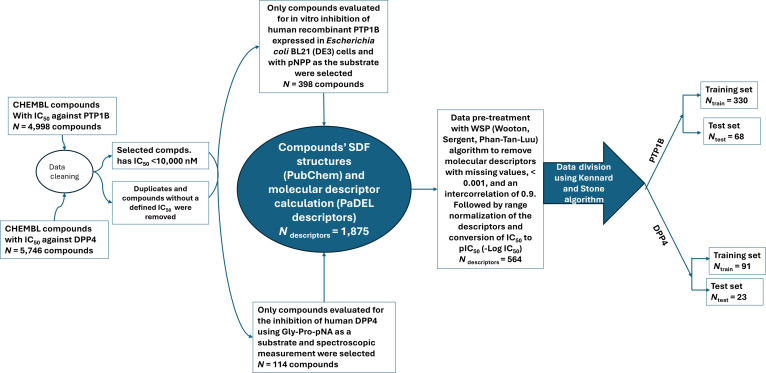
Schematic workflow for the data collection, curation, and partitioning of bioactive compounds used in the development of the PTP1B and DPP4 QSAR models.

In this study, a QSAR model was developed to predict the PTP1B and DPP4 inhibitory activities of the identified bioactive compounds. The modeling framework utilized a genetic algorithm (GA) for optimal feature selection and multiple linear regression (MLR) for predictive construction. Figure 1 illustrates the chemical composition and sources of the compounds employed in the model training and validation process. A total of 398 compounds with in vitro inhibitory activity against human recombinant PTP1B expressed in *Escherichia coli* BL21 (DE3) cells, using pNPP as the substrate, were used for training and testing the PTP1B QSAR model. One hundred fourteen compounds with inhibitory activity against human DPP4, using Gly-Pro-pNA as a substrate, were employed for constructing the DPP4 QSAR model. The compounds’ molecular structures were obtained from the PubChem database and optimized using the PM3 semi-empirical quantum mechanical method in Spartan’18 software. These were then used to calculate approximately 1,875 molecular descriptors using the PaDEL_Descriptor software. After rigorous data preprocessing, an *n* (398) by *m* (564) matrix was obtained for PTP1B, and an *n* (114) by *m* (564) matrix was obtained for DPP1, which served as the dataset, where *n* is the number of compounds and *m* is the number of descriptors in the dataset. The Kennard and Stone algorithm available in Data_Division 1.2 (DTC lab) split the PTP1B dataset into a 330-training set and a 68-test set, and the DPP4 dataset into a 91-training set and a 23-test set. GA 1.3 (DTC lab) was used to select the most appropriate descriptor, with the following parameter settings: total number of iterations/generations (1,000), equation length (8), crossover probability (1), mutation probability (0.5), initial number of equations generated (100), and number of equations selected in each generation (3). The best group of descriptors, based on internal validation parameters, was selected for both the training and test sets. MLRPlusValidation1.3 (DTC lab) was then used to build the model.

The QSAR model was validated both internally and externally to ensure robustness and predictive reliability. Internal validation was performed using leave-one-out (LOO) cross-validation, with the cross-validated coefficient of determination (Q^2^_LOO_) quantifying the accuracy of pIC_50_ predictions. External validation utilized the test set compounds. The consistency and predictive capability of the model were evaluated by calculating the mean absolute error (MAE) for the test set. Furthermore, external predictability was rigorously assessed against the statistical criteria established in literature [27]: A QSAR model is considered predictive if the following conditions are satisfied:

I. *Q*^2^ > 0.5
II. *R*^2^ > 0.6
III. *R*^2^ − *R*^2^_0_/*R*^2^ < 0.1 and 0.85 ≤ *k* ≤ 1.15 OR *R*^2^ − *R′*^2^_0_/*R*^2^ < 0.1 and 0.85 ≤ *k′* ≤ 0.15
IV. │*R*^2^_0_ − *R′*^2^_0_│ < 0.3
V. *R*^2^ or *R′*^2^_0_ close to *R*^2^

where *R*^2^ and *R*^2^_0_ are the square correlation between the experimental pIC_50_ and the QSAR model predicted pIC_50_ with and without intercept, respectively, while *R*′^2^_0_ represents the same information as *R*^2^_0_ does but with inverted axis (linear regression between the predicted against the observed values). The above parameters can be calculated using the following equations:

$$R_{0}^{2}=1-\frac{\sum {\left(Y_{\text{pred}}-Y_{\mathrm{obs}}^{\prime 0}\right)}^{2}}{\sum {\left(Y_{\text{pred}}-{\bar{Y}}_{\text{pred}}\right)}^{2}}$$ (1)

$$R_{0}^{\prime 2}=1-\frac{\sum {\left(Y_{\mathrm{obs}}-Y_{\text{pred}}^{\prime 0}\right)}^{2}}{\sum {\left(Y_{\mathrm{obs}}-{\bar{Y}}_{\mathrm{obs}}\right)}^{2}}$$ (2)

where ${\bar{Y}}_{\text{pred}}$ and ${\bar{Y}}_{\mathrm{obs}}$ refer to the mean values of the predicted and observed activity data, respectively. The regression lines through the origin are defined by *Y*^*r*0^*_obs_* = *k Y_pred_* and *Y*^*r*0^*_pred_* = *k*′ *Y_obs_*, while the slopes *k* and *k′* are calculated as follows:

$$k=\frac{\sum Y_{\mathrm{obs}}Y_{\text{pred}}}{\sum Y_{\text{pred}}^{2}}$$ (3)

$$k^{\prime }=\frac{\sum Y_{\mathrm{obs}}Y_{\text{pred}}}{\sum Y_{\mathrm{obs}}^{2}}$$ (4)

Likewise, *R*^2^_m_ metrics proposed in literature [28] for external validation were calculated to further evaluate the correlation between the observed and predicted activity:

$${\bar{R}}_{m}^{2}=\frac{\left(R_{m}^{2}+R_{m}^{\prime 2}\right)}{2}$$ (5)

where $\begin{array}{c} R_{m}^{2}=R^{2}\times \left(1-\sqrt{R^{2}-R_{0}^{2}}\right) \end{array}$ and $\begin{array}{c} R_{m}^{\prime 2}=R^{2}\times \left(1-\sqrt{R^{2}-R_{0}^{\prime 2}}\right) \end{array}$

Furthermore, the QSAR model applicability domain (AD), which represents the theoretical region in the chemical space defined by the descriptors and response variable of the training set, within which a model can make reliable predictions, was developed with the Williams plot method using Microsoft Excel. This also provides a simultaneous visualization of the structural and response space by plotting the standardized residuals against the leverage values (*h*). The leverage (*hi*) for each compound *i* was calculated using the Hat matrix (**H**) as follows:

$$H=X{\left(X^{T}X\right)}^{-1}X^{T}$$ (6)

where **X** represents the descriptor matrix of the training set. Warning leverage (h*) was established as the threshold for the structural domain, calculated as follows:

$$h^{*}=\frac{3p}{n}$$ (7)

​where *p* is the number of model descriptors plus one and *n* is the number of training compounds. Compounds with *h* > h* were identified as structural outliers. Furthermore, the response domain was bounded by a threshold of ±3 standard deviation units for the standardized residuals. Compounds falling within both the warning leverage (h*) and the residual limits (±3) were within the AD of the model [29].

## Results and Discussion

### Molecular docking results and binding poses

**Fig. 2.**
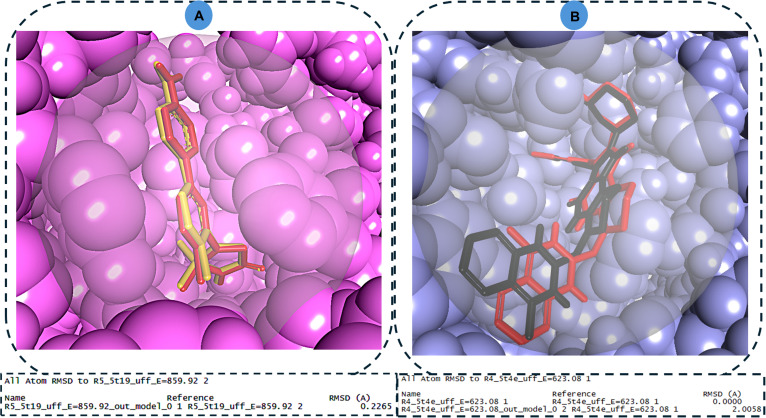
Superimposition of the auto-dock-derived ligand pose over the crystallographic pose obtained from PDB for (A) PTP1B, RMSD = 0.226 Å, and (B) DPP4, RMSD = 2.005.

The molecular docking protocol was validated by superimposing docked co-crystallized ligands onto their experimental configurations retrieved from PDB, yielding RMSD values of 0.23 Å for PTP1B and 2.0 Å for DPP4 (Fig. 2). In general, RMSD quantifies 3D positional deviation between corresponding atoms in the predicted (docked) and reference crystallographic structures [30,31]. While the 2.0-Å value for DPP4 represents the upper threshold of acceptance [5,32], it is primarily attributed to the limitations of the rigid-receptor docking algorithm used in this study, which does not account for side-chain rearrangements or induced-fit adjustments during the initial search. The DPP4 binding pocket is characterized by significant conformational plasticity and a solvent-exposed nature, which often requires side-chain flexibility for optimal ligand positioning [33]. To overcome the limitations of static, rigid docking and to allow for these necessary side-chain rearrangements, all complexes were subjected to 120-ns MD simulations. This post-docking refinement ensured that the reported interactions reflect a relaxed, dynamic state where the protein–ligand interface has undergone induced-fit adjustments, providing a more biologically relevant representation of the binding affinity.

Utilizing the validated docking protocol, 5 lead compounds were prioritized for each target based on their superior binding affinity scores (Table 1) from a total of 378 compounds screened (Table S1). The selected compounds demonstrated a more favorable docking score for PTP1B relative to ursolic acid (−6.10 kcal/mol). Rutin exhibited a docking score of −9.60 kcal/mol, demonstrating a binding affinity for DPP4 comparable to the reference inhibitor sitagliptin (−9.40 kcal/mol) within the inherent precision limits of the scoring function. Similarly, quercetin-3,7-diglucoside (Q37DG) and quercetin-7-O-glucoside (Q7G) also demonstrated favorable binding profiles (Table 1). These qualitative rankings suggest that the identified flavonoid glycosides may serve as multi-target agents in type 2 diabetes management by inhibiting PTP1B-mediated dephosphorylation and DPP4-driven incretin degradation [34]. The high binding affinity observed for these glycosylated flavonoids is consistent with the reported inhibitory activity of hespereretin-5-O-glucoside against PTP1B reported in the literature [35], demonstrating the critical role of flavonoid glycosylation in modulating potency against this anti-diabetic target. Furthermore, flavonoids hyperoside, narcissoside, cyaniding 3-O-glucoside, and isoliquiritigenin had been reported to show favorable binding in the active site of DPP4 [35].

**Table 1.** Structures and docking score of the top five computationally identified compounds and reference standards against PTP1B and DPP4.

PTP1B		DPP4	
Structure	Docking Score (kcal/mol)	Structure	Docking Score (kcal/mol)
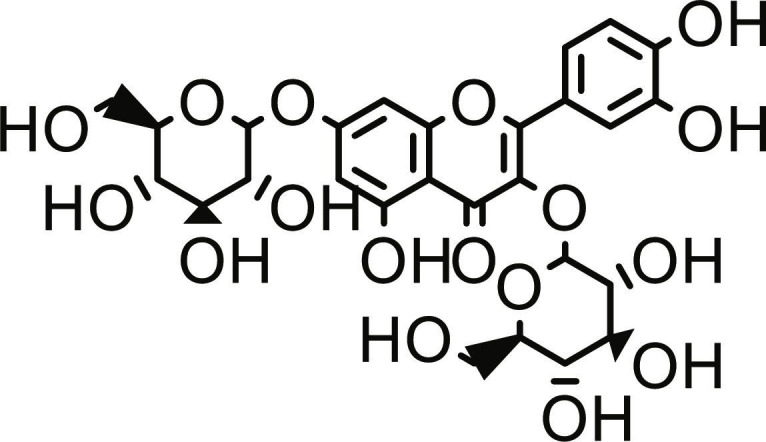 Quercetin-3,7-diglucoside	−9.00	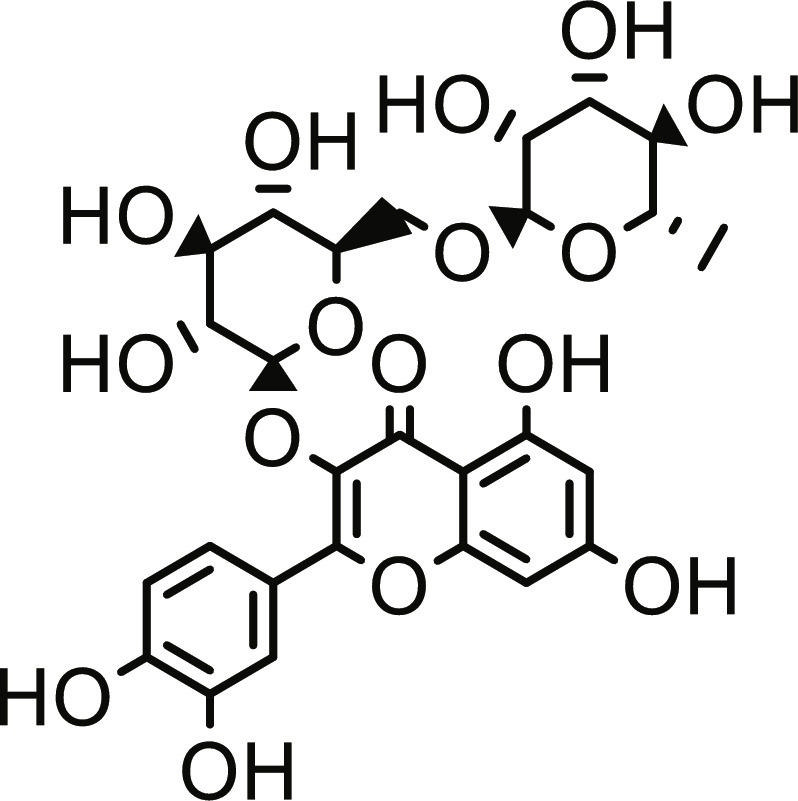 Rutin	−9.60
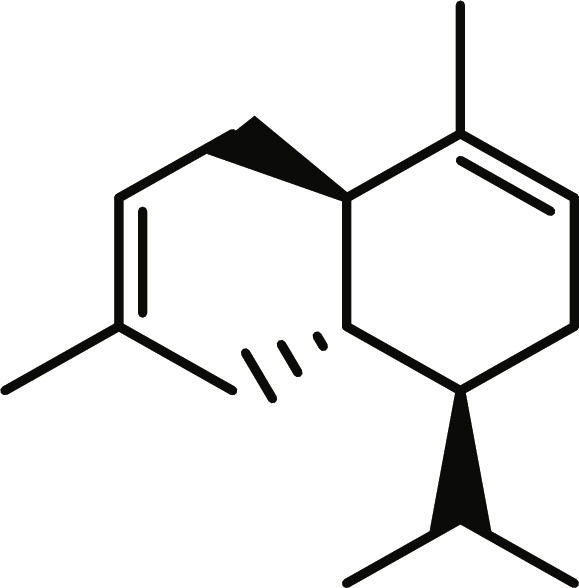 Cadinene	−8.70	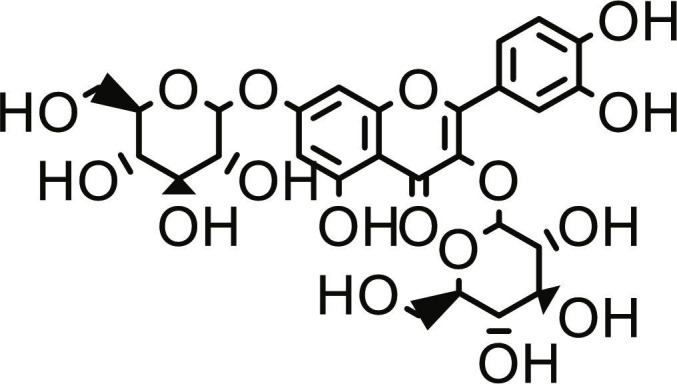 Quercetin-3,7-diglucoside	−9.20
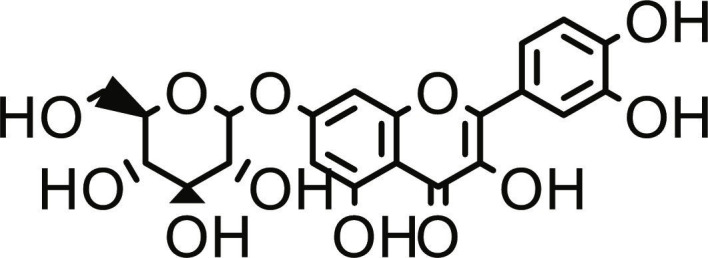 Quercetin-7-O-glucoside	−8.50	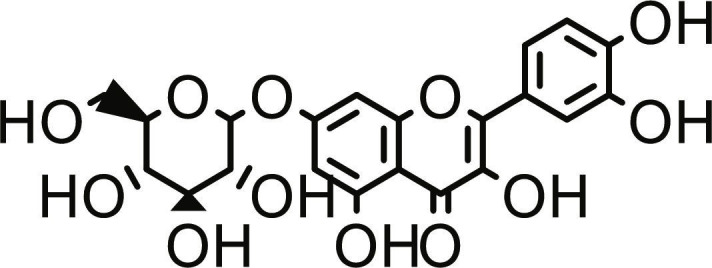 Quercetin-7-O-glucoside	−9.20
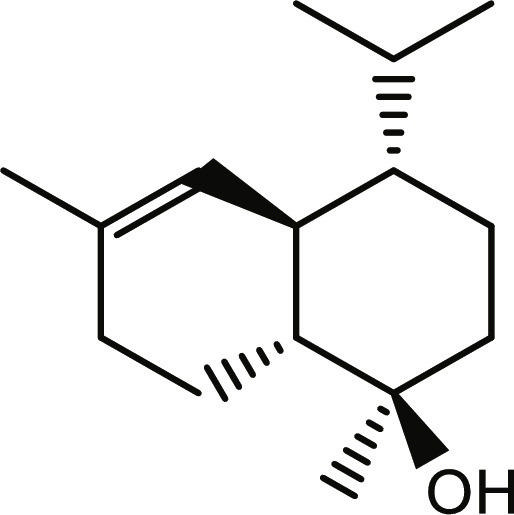 Cadinenol	−8.50	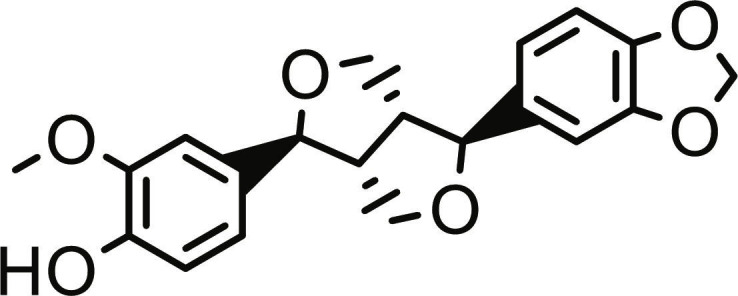 Piperitol	−8.50
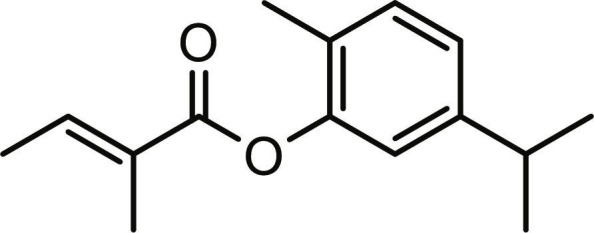 5-isopropy-2-methylphenyl-2-methylbutyl-2-ethanoate	−8.40	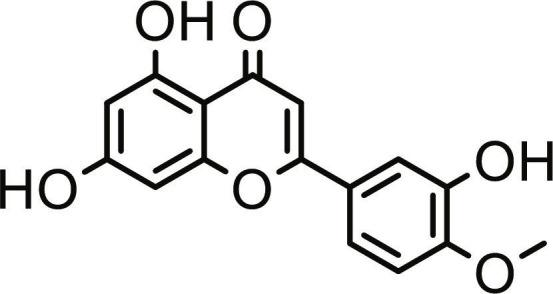 Diosmetin	−8.30
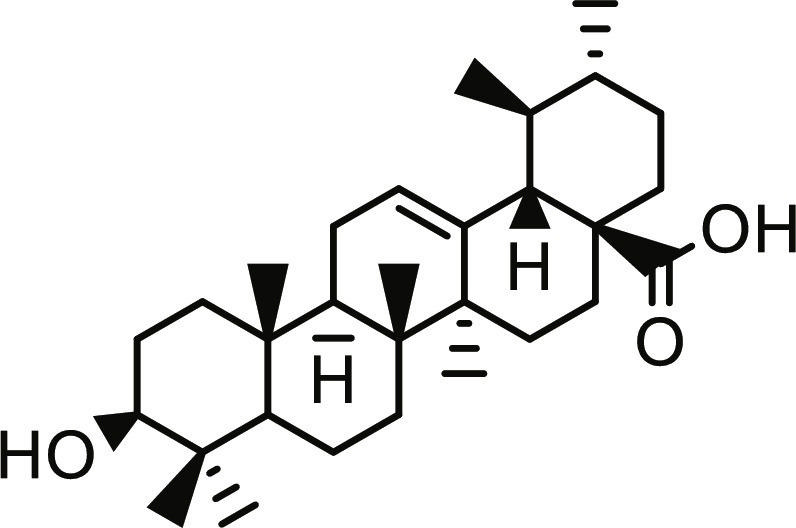 Ursolic acid	−6.10	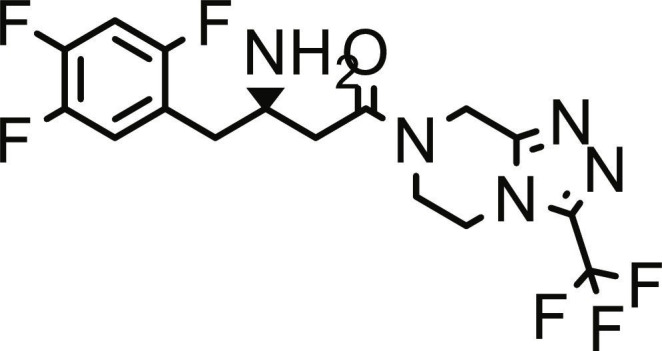 Sitagliptin	−9.40

### MMGBSA binding free energy analysis

Static docking often fails to capture the dynamic conformational changes and solvation effects of protein–ligand interactions; however, MD MMGBSA can account for these effects [36]. To evaluate the relative binding affinity of the prioritized compounds, a 120-ns MD simulation was done on the complexes of the selected top 5 compounds with the enzymes. The analysis of the post-dynamic simulation result via MMGBSA methods showed that for PTP1B complexes, Q37DG (Δ*G*_bind_ = −52.19 ± 8.31 kcal/mol) and Q7G ( Δ*G*_bind_ = −38.63 ± 7.78 kcal/mol) demonstrated more favorable relative binding energy scores compared to ursolic acid (Δ*G*_bind_ = −24.95 ± 4.39 kcal/mol) (Table 2). This corroborates their observed docking scores and suggests a robust binding profile within the computational framework. It is important to note that the calculations did not account for configurational entropy contributions, which often lead to the observation of exceptionally large negative values. The magnitude (ranging from −52 to −58 kcal/mol) is characteristic of the MMGBSA method when applied to highly polar and multi-glycosylated ligands, where the continuum solvent model tends to overestimate binding due to dominant electrostatic and van der Waals terms. Consequently, these scores are interpreted here as relative indicators of complex stability and qualitative ranking tools rather than absolute physical thermodynamic free energy.

**Table 2.** Thermodynamic energy (kcal/mol) profile of selected top 5 compounds for PTP1B and DPP4

Complexes	Δ*Ε*_vdW_	Δ*E*_elec_	Δ*E*_GB_	Δ*E*_surf_	Δ*G*_gas_	Δ_solv_	Δ*G*_bind_
PTP1B							
PTP1B–Q37DG	−44.05 ± 7.74	−94.84 ± 23.2	93.93 ± 14.38	−7.22 ± 0.50	−138.9 ± 19.8	86.71 ± 14.2	−52.19 ± 8.31
PTP1B–β-cadinene	−13.82 ± 7.87	−0.44 ± 0.95	4.96 ± 2.41	−1.84 ± 1.03	−14.27 ± 8.04	3.12 ± 1.55	−11.14 ± 6.94
PTP1B–Q7G	−39.39 ± 6.84	−55.55 ± 17.3	61.92 ± 11.06	−5.39 ± 0.62	−94.95 ± 15.4	56.32 ± 11.1	−38.63 ±7.78
PTP1B–cadinenol	−12.21 ± 6.78	−2.59 ± 3.80	7.49 ± 4.59	−1.62 ± 0.90	−14.80 ± 8.80	5.86 ± 3.94	−8.94 ± 5.76
PTP1B–5IMME	−16.76 ± 6.97	−5.93 ± 4.10	10.13 ± 4.67	−2.32 ± 0.98	−22.70 ± 9.87	7.76 ± 3.90	−14.93 ± 6.78
PTP1B–ursolic acid	−31.48 ± 4.76	−5.38 ± 7.66	15.93 ± 5.49	−4.00 ± 0.57	−36.87 ± 7.75	11.92 ± 5.39	−24.95 ± 4.39
DPP4							
DPP4–rutin	−38.25 ± 5.19	−87.7 ± 20.3	82.42 ± 15.50	−5.82 ± 0.37	−125.9 ± 18.7	76.59 ± 15.4	−49.36 ± 6.55
DPP4–Q37DG	−44.41 ± 5.94	−103.3± 23.3	96.54 ± 12.34	−6.98 ± 0.57	−147.7 ± 22.9	89.55 ± 12.0	−58.23 ± 14.1
DPP4–Q7G	−28.73 ± 4.89	−81.53 ± 18.7	74.81 ± 10.84	−4.50 ± 0.36	−110.9 ± 16.6	70.31 ± 10.7	−39.95 ± 7.58
DPP4–piperitol	−26.55 ± 4.09	−22.12 ± 13.4	31.90 ± 7.93	−3.37 ± 0.46	−48.68 ± 13.2	28.60 ± 7.75	−20.07 ± 6.75
DPP4–diosmetin	−30.66 ± 4.98	−24.55 ± 7.96	35.89 ± 5.99	−3.77 ± 0.39	−55.22 ± 8.49	32.12 ± 5.89	−23.11 ± 4.53
DPP4–sitagliptin	−26.17 ± 5.93	−315.3 ± 43.2	321.49 ± 39	−4.02 ± 0.57	−341.5 ± 39	317.4 ± 38.5	−24.10 ± 6.91

Q7G, quercetin-7-O-glucoside; Q37DG, quercetin-3,7-diglucoside; 5IMME, 5-isopropy-2-methylphenyl-2-methylbutyl-2-ethanoate; Δ*Ε*_vdW_, change in van der Waals energy; Δ*E*_elec_, change in electrostatic energy; Δ*E*_GB_, change in generalized Born solvation energy; Δ*E*_surf_, change in surface area energy; Δ*G*_gas_, change in gas phase free energy; Δ*G*_solv_, change in solvation free energy; Δ*G*_bind_, total binding free energy

Similarly, MMGBSA analysis showed that Q37DG, Q7G, and rutin exhibited more favorable relative binding profiles for DPP4 when compared to sitagliptin, which showed a binding free energy of (Δ*G*_bind_ = −24.10 ± 6.91 kcal/mol) (Table 2). While this observation aligned with the docking result for rutin, there is a clear discrepancy in the Q37DG and Q7G results, which underscores the unreliability of docking analysis alone [37]. Therefore, since sitagliptin is an established DPP4 inhibitor, Q37DG, Q7G, and rutin could be regarded as potential DPP4 inhibitors prioritized for further experimental validation.

Notably, Q37DG, Q7D, and rutin (the compounds with significant favorable binding for both PTP1B and DPP4) are major metabolites identified through data mining of the phytochemical profile of *A. betulina*. Their inclusion in this study provides scientific evidence for the observed traditional antidiabetic properties of *A. betulina* [7] and offers insights into the potential inhibitory contributions of the plant’s broader secondary metabolite matrix. Analysis of the energetics of enzyme–ligand interaction in this study via correlation analysis (Table S2) showed that the binding of the compounds with the enzyme is predominantly governed by van der Waals interaction, which had a correlation coefficient *r* of 0.924 with the binding free energy Δ*G*_bind_.

### Post-MD: Integrative stability and convergence analyses

**Fig. 3.**
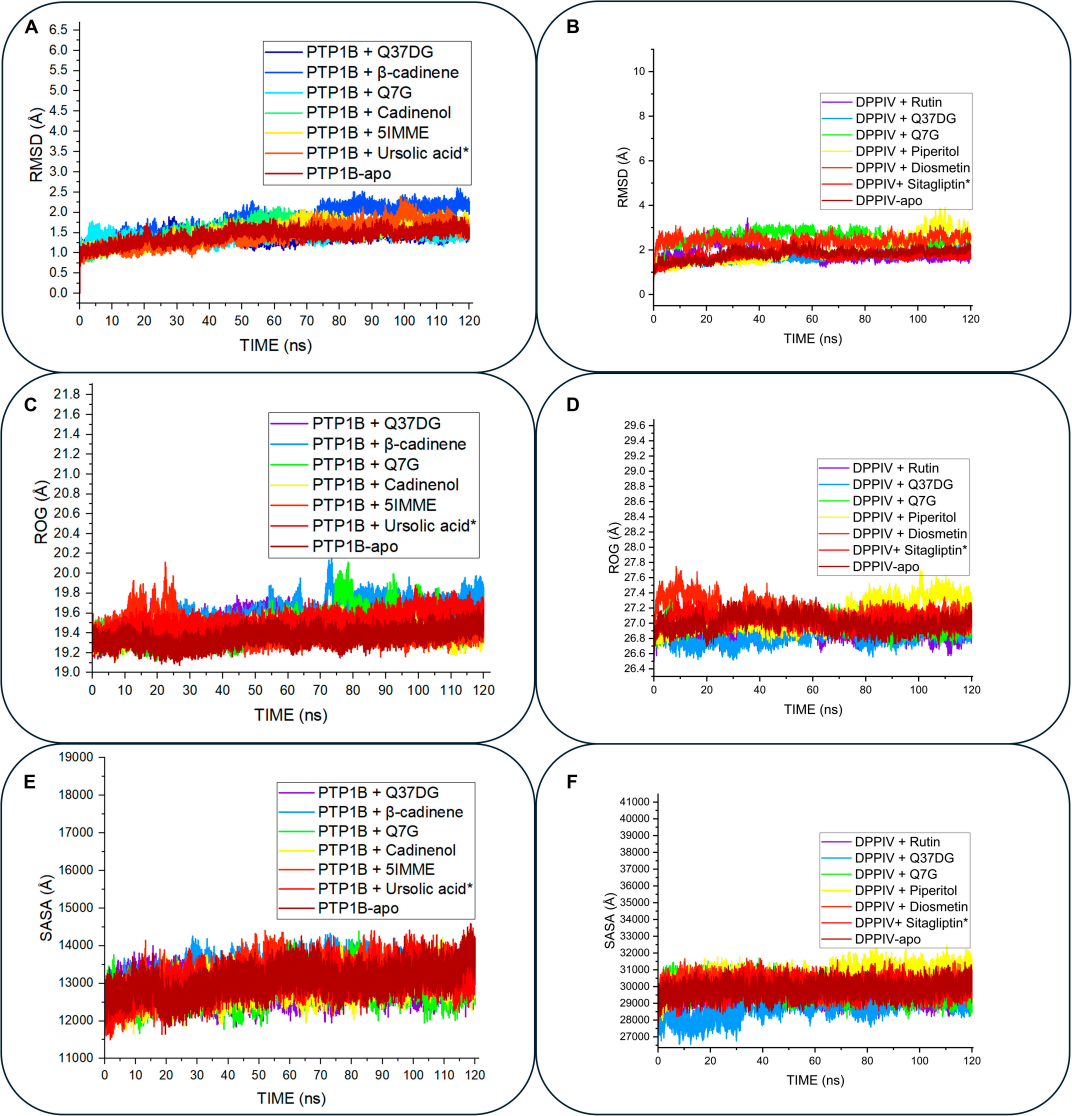
Post-MD simulation parameters time evolution trajectories plot for both PTP1B and DPP4 antidiabetic targets. RMSD plots revealed that Q37DG maintains the lowest fluctuation range between roughly 1.5 and 2.0 Å in both PTP1B (A) and DPP4 (B), which indicates superior stability when compared to other compounds and reference compounds (ursolic acid and sitagliptin). The ROG plots also showed Q37DG line progressing slightly above the apo line for PTP1B (C) and below that of the apo and other compounds for DPP4 (D), revealing its capability to preserve compact protein–ligand structure throughout MD simulation time. The SASA plot progressed in a similar pattern to that of RMSD for PTP1B (E) and DPP4 (F), showing that Q37DG complexes maintain a stable SASA necessary for binding. This analysis portrays Q37DG as a potential lead for multi-target antidiabetic therapy.

The 120-ns MD trajectories were analyzed to assess the structural integrity and convergence of the enzyme–ligand complexes, revealing a clear distinction between the behavior of polar flavonoid glycosides and volatile terpenes. The structural stability of the complexes was primarily evaluated through RMSD [38]. The flavonoid leads (Q37DG, rutin, and Q7G) and reference standards achieved rapid equilibration within 25 ns (Fig. 3A and B), maintaining average RMSD values (1.43 to 2.52 Å) for both targets, which mirrors the apo-enzyme’s stability (Table 3). Biologically, this indicates that these leads occupy the catalytic pocket without inducing unfavorable conformational strain. Conversely, a significant “biologically meaningful deviation” was observed for the volatile constituents, β-cadinene and cadinenol; their trajectories exhibited continuous drift and erratic fluctuations (up to 2.5 Å), suggesting that these smaller, hydrophobic molecules fail to form a stable residence within the polar binding sites of PTP1B and DPP4.

**Table 3.** Post-dynamic parameters and correlations with binding free energy for PTP1B and DPP4 complexes

Complex systems		RMSD (Å)	ROG (Å)	RMSF (Å)	SASA (Å^2^)	H-bond
PTP1B					
PTP1B–Q37DG		1.48 ± 0.11	19.49 ± 0.08	1.06 ± 0.49	12,930.30 ± 270.93	156.57 ± 8.07
PTP1B–β-cadinene		1.50 ± 0.34	19.58 ± 0.13	1.20 ± 0.56	13,227.25 ± 325.12	152.09 ± 8.48
PTP1B–Q7G		1.45 ± 0.12	19.45 ± 0.14	1.04 ± 0.53	12,898.03 ± 343.23	153.45 ± 8.36
PTP1B–cadinenol		1.46 ± 0.24	19.41 ± 0.06	1.15 ± 0.50	12,933.48 ± 338.33	151.61 ± 8.82
PTP1B–5IMME		1.44 ± 0.24	19.42 ± 0.11	1.10 ± 0.56	13,157.67 ± 402.71	152.92 ± 8.02
PTP1B–ursolic acid		1.43 ± 0.29	19.50 ± 0.09	1.03 ± 0.55	13,078.24 ± 353.52	152.94 ± 8.18
PTP1B–apo		1.41 ± 0.17	19.35 ± 0.07	1.08 ± 0.54	13,047.79 ± 356.31	151.22 ± 8.58
DPP4						
DPP4–rutin		1.84 ± 0.33	26.90 ± 0.08	1.05 ± 0.51	29,503.69 ± 401.15	380.35 ± 12.7
DPP4–Q37DG		1.67 ± 0.19	26.83 ± 0.09	1.15 ± 0.47	28,926.93 ± 694.05	386.70 ± 13.5
DPP4–Q7G		2.52 ± 0.33	27.00 ± 0.09	1.16 ± 0.50	29,870.35 ± 444.01	380.30 ± 12.3
DPP4–piperitol		1.98 ± 0.57	27.10 ± 0.17	1.17 ± 0.49	30,536.28 ± 546.52	373.55 ± 12.8
DPP4–diosmetin		2.41 ± 0.20	27.16 ± 0.13	1.17 ± 0.47	30,007.78 ± 403.86	374.01 ± 12.3
DPP4–sitagliptin		1.71 ± 0.17	27.04 ± 0.08	1.14 ± 0.45	29,943.96 ± 416.13	381.91 ± 13.6
DPP4–apo		1.81 ± 0.22	27.00 ± 0.09	1.15 ± 0.52	29,805.72 ± 383.89	378.13 ± 12.3
Correlation matrix						
Parameters	Δ*G*_bind_	RMSD (Å)	ROG (Å)	RMSF (Å)	SASA (Å)	H-bond
PTP1B						
Δ*G*_bind_	1.000					
RMSD	−0.036	1.000				
ROG	−0.059	0.575	1.000			
RMSF	0.830	0.444	0.415	1.000		
SASA	0.594	0.188	0.508	0.659	1.000	
nH-bond	−0.923	0.187	0.083	−0.698	−0.406	1.000
DPP4						
Δ*G*_bind_	1.000					
RMSD	0.311	1.000				
ROG	0.952	0.549	1.000			
RMSF	0.519	0.373	0.538	1.000		
SASA	0.918	0.411	0.888	0.409	1.000	
nH-bond	−0.789	−0.545	−0.885	−0.303	−0.864	1.000

The formation of rigid, well-folded complexes was further corroborated by the synchronization of ROG (Fig. 3C and D) [39] and SASA parameters (Fig. 3E and F) [40]. For both targets, the flavonoid leads maintained lower and more consistent Rg and SASA values compared to the volatile compounds. Notably, Q37DG demonstrated the most significant surface burial in the DPP4 pocket (SASA: 28,926.93 Å^2^ versus Apo: 29,805.72 Å^2^) (Table 3). This reduction in SASA, coupled with a stable ROG profile, confirms that the protein–ligand interface is highly compact, effectively shielding the catalytic residues from the solvent and stabilizing the inhibited state.

**Fig. 4.**
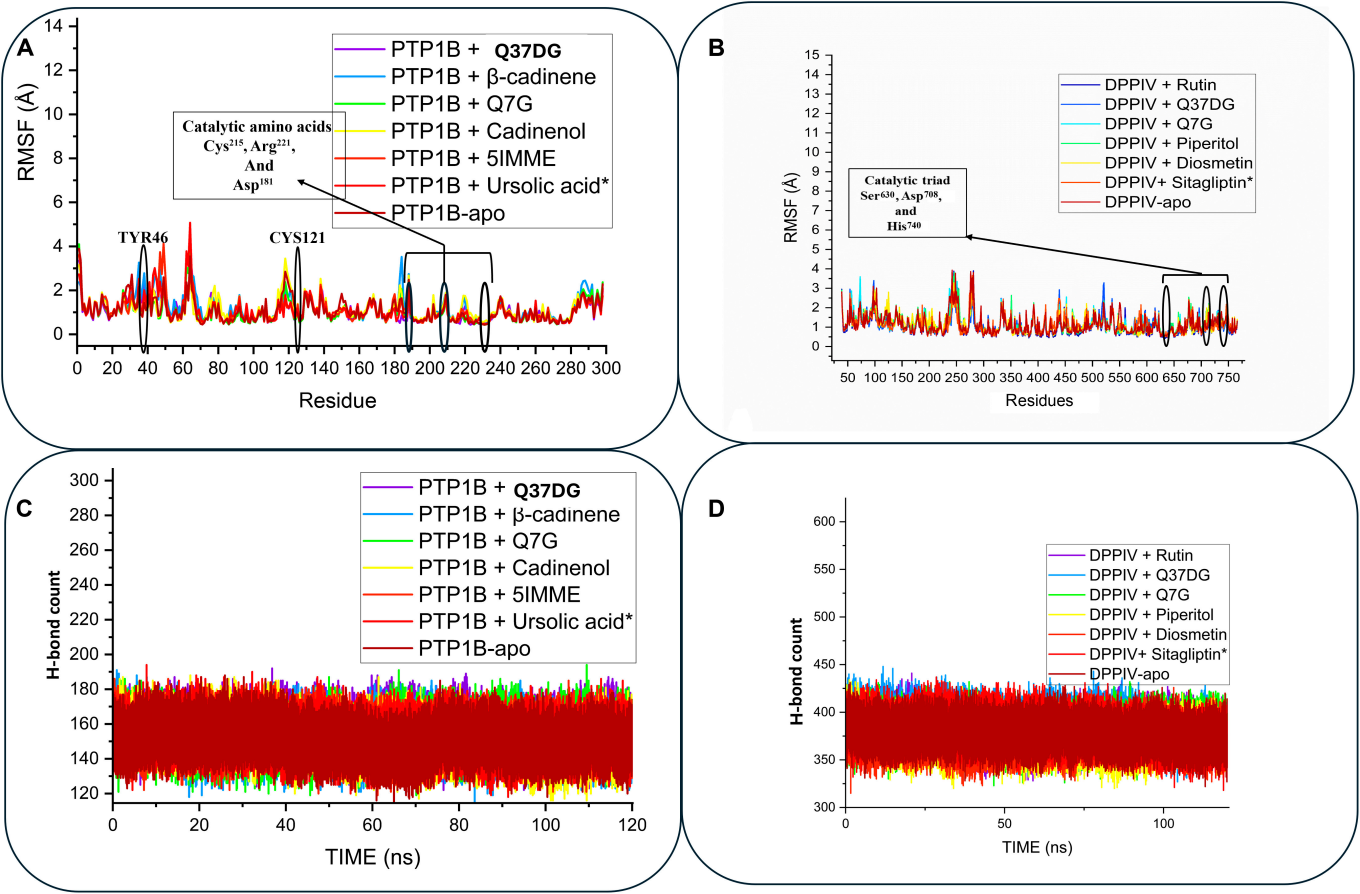
Post-MD parameter trajectories evolution plot over 120-ns simulation time. (A) RMSF plot for PTP1B showed that Q37DG demonstrated the lowest RMSF progression in relation to other compounds, which is indicative of a reduction in protein flexibility after binding to the ligand. (B) RMSF plots for DPP4 complexes showing Q37DG and rutin with stable and low fluctuation in relation to other compounds, which indicate favorable binding. (C and D) The hydrogen bond count plot displayed consistently similar progression for all the compounds throughout the simulation period. The result supported the potential of Q37DG as a lead for antidiabetic treatment.

The structural rigidity observed in the RMSD and ROG analyses is functionally driven by a robust hydrogen bond network. A strong correlation (r=−0.923) (Table 3) was identified between the frequency of hydrogen bonds and the calculated binding free energies. These integrative data confirm that the superior stability of Q37DG and Q7G is not only a result of geometric fit but also a consequence of sustained electrostatic and van der Waals interactions that stabilize the catalytic regions. These findings identify Q37DG as the standout lead for dual-target stabilization, providing the structural sustained rigidity required for effective antidiabetic therapy (Fig. 4).

### Enzyme–ligand interaction profile analysis

**Fig. 5.**
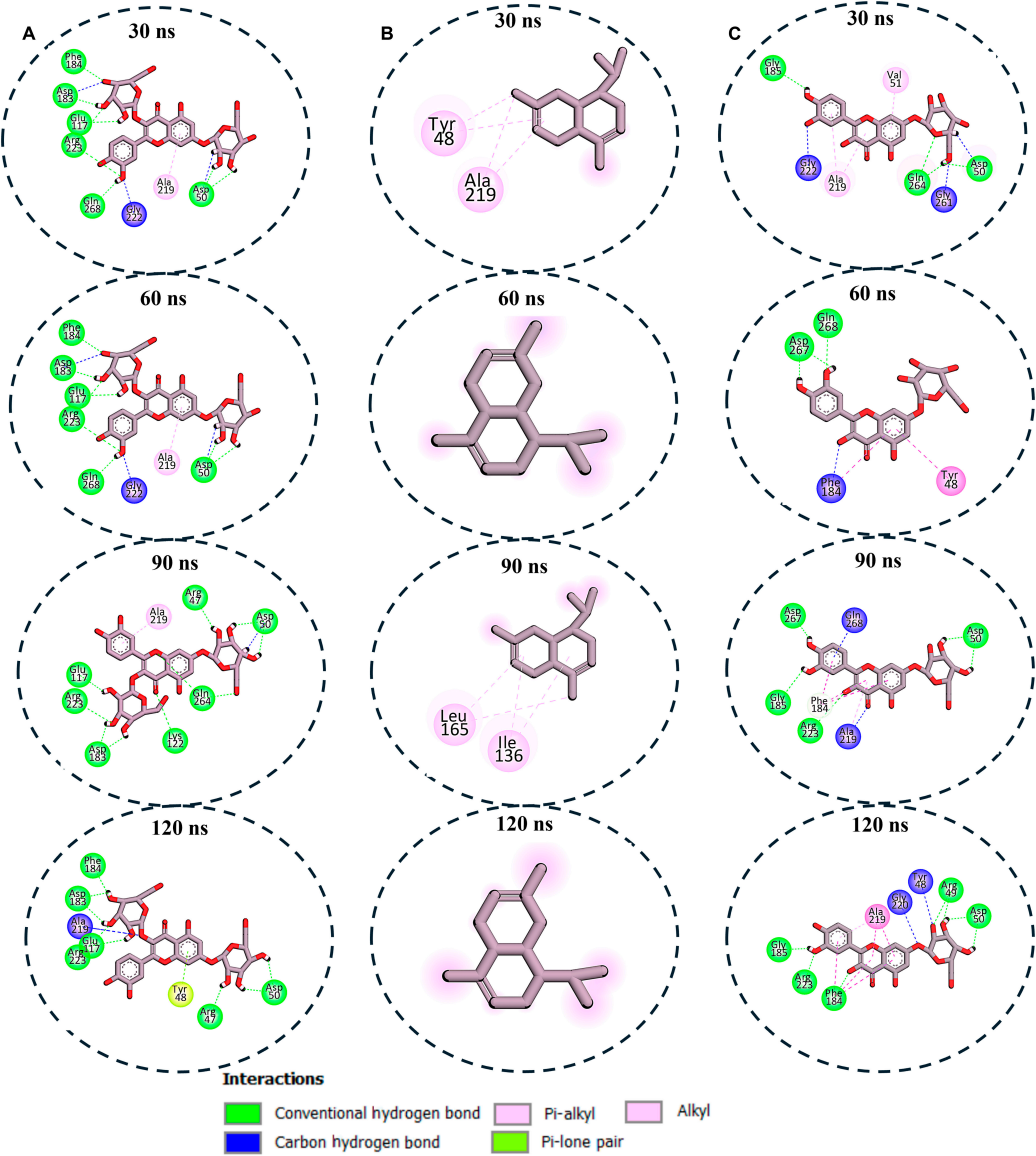
Enzyme–ligand interaction analysis over 120-ns MD simulation for the (A) PTP1B–Q37DG interaction, which showed that the compound retains steady interaction with 8 to 12 active site amino acid residues during the simulation, making it the compound with exceptional binding stability, which is predominantly mediated by hydrogen bonding and hydrophobic contacts. (B) The PTP1B–cadinene system, on the other hand, left the active site after 30 ns of simulation time, which could be responsible for its low binding free energy. (C) The PTP1B–Q7G complex also had consistent interaction with 4 to 8 amino acid residues during the simulation, which indicates intermediate binding to the enzyme when compared to that of Q37DG.

To gain insight into the interaction between the compounds and the studied enzyme, MD snapshots were taken at different time intervals, and their interaction profiles were analyzed using Discovery Studio Visualizer. This was done to provide support for the variation in the binding affinities calculated for the different compounds at the molecular level. The interaction plots for the PTP1B systems showed that the exceptional stability of Q37DG, which is exemplified by its high binding free energy (Table 1), could be attributed to its consistent interactions with 11 critical residues (Arg^47^, Tyr^48^, Asp^50^, Glu^117^, Lys^122^, Asp^183^, Phe^184^, Ala^219^, Gly^222^, Arg^223^, and Gln^268^) throughout the simulation via multiple interaction types (Fig. 5A). This aligned closely with established literature regarding the inhibition of PTP1B by flavonoids. PTP1B is a well-characterized target for type 2 diabetes, and its catalytic activity is governed by specific structural motifs: the P-loop (residues 214 to 221), the tryptophan (W), proline (P), and apartic acid (D) WPD loop (residues 179 to 187), and the glutamine (Q) Q-loop (residues 260 to 262). The interaction of Q37DG with Asp^183^ (within the WPD loop), which acts as a general acid/base catalyst, and Arg^223^ (within the P-loop), which is critical for stabilizing the phosphate group of substrates, is an established way flavonoids like quercetin and its glycosides mimic PTP1B substrate interactions. Furthermore, “eleven critical residues” identified for Q37DG suggest a multi-site binding mode that likely bridges the active site and the secondary aryl-phosphate binding site (Site B), a strategy often cited in literature to enhance selectivity over other phosphatases like TCPTP [34,41–43].

**Fig. 6.**
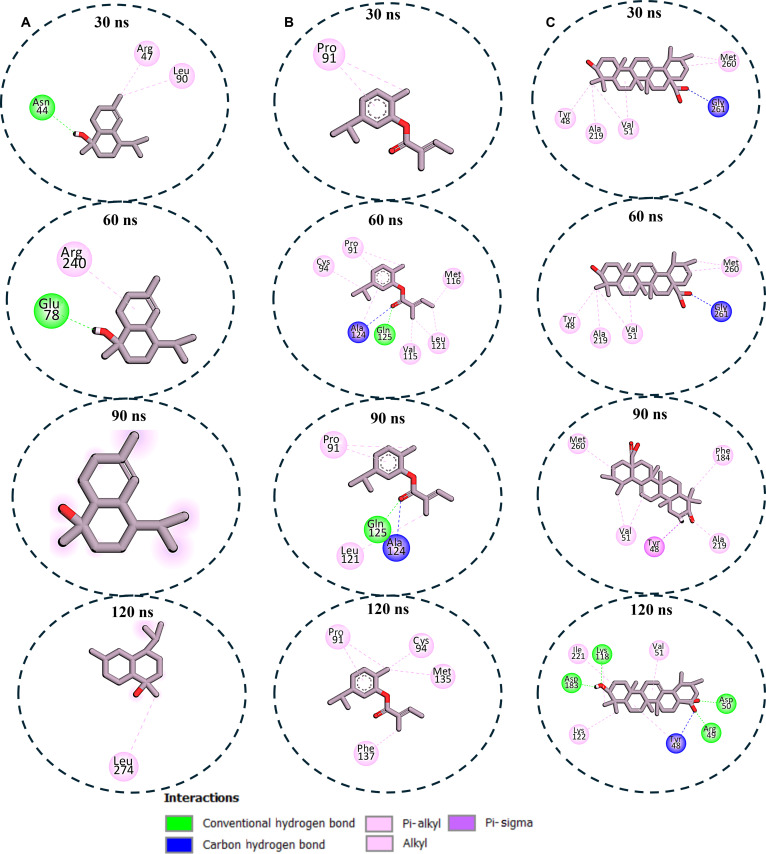
Enzyme–ligand interaction analysis over 120-ns MD simulation for (A) PTP1B–cadinenol complex interaction showing unstable interaction between the enzyme and the ligand, and at 90 ns, the ligand left the binding, which indicates poor binding potential. (B) The PTP1B–5IMME complex system showed an increment in the number of amino acid residues interacting with the ligand as time progressed, and at the end of the simulation, the compound retained binding with 4 amino acid residues via hydrophobic interactions, suggesting moderate inhibitory potential. (C) The PTP1B–ursolic acid complex system displayed stable binding throughout the MD simulation time via both hydrogen bonding and hydrophobic interaction, which indicates potential superior binding affinity relative to the other 2 compounds.

In contrast, β-cadinene exhibited transient binding with Tyr^48^ and Ala^219^ at 30 ns before complete dissociation by 60 ns, which means unfavorable thermodynamic profiles agreeing with the calculated binding free energy for this compound (Fig. 5B). The complete dissociation observed is a well-documented limitation of simple sesquiterpenes in the PTP1B polar catalytic pocket [44,45]. Like Q37DG, Q7G displayed intermediate stability with dynamic interactions involving key residues Asp^50^, Tyr^48^, Phe^184^, Ala^219^, Arg^223^, and Gln^264^, fluctuating between 4 and 8 contacts across timeframes (Fig. 5C). Cadinenol also showed highly unstable binding, transiently engaging Asn^44^, Arg^47^, and Leu^90^ before complete dissociation at 90 ns (Fig. 6A). 5IMME demonstrated progressive stabilization, expanding from a single Pro^91^ contact to multiple residue engagement, including catalytically crucial Cys^94^ (Fig. 6B). The reference ursolic acid also showed a significant interaction profile, establishing consistent interactions with Tyr^48^, Val^51^, Asp^183^, and Ala^219^, with binding interface optimization incorporating 8 residues at 120 ns (Fig. 6C). In conclusion, the complexity of the numbers and types of interaction between the enzyme and Q37DG and Q7G validates them as potential PTP1B inhibitors.

**Fig. 7.**
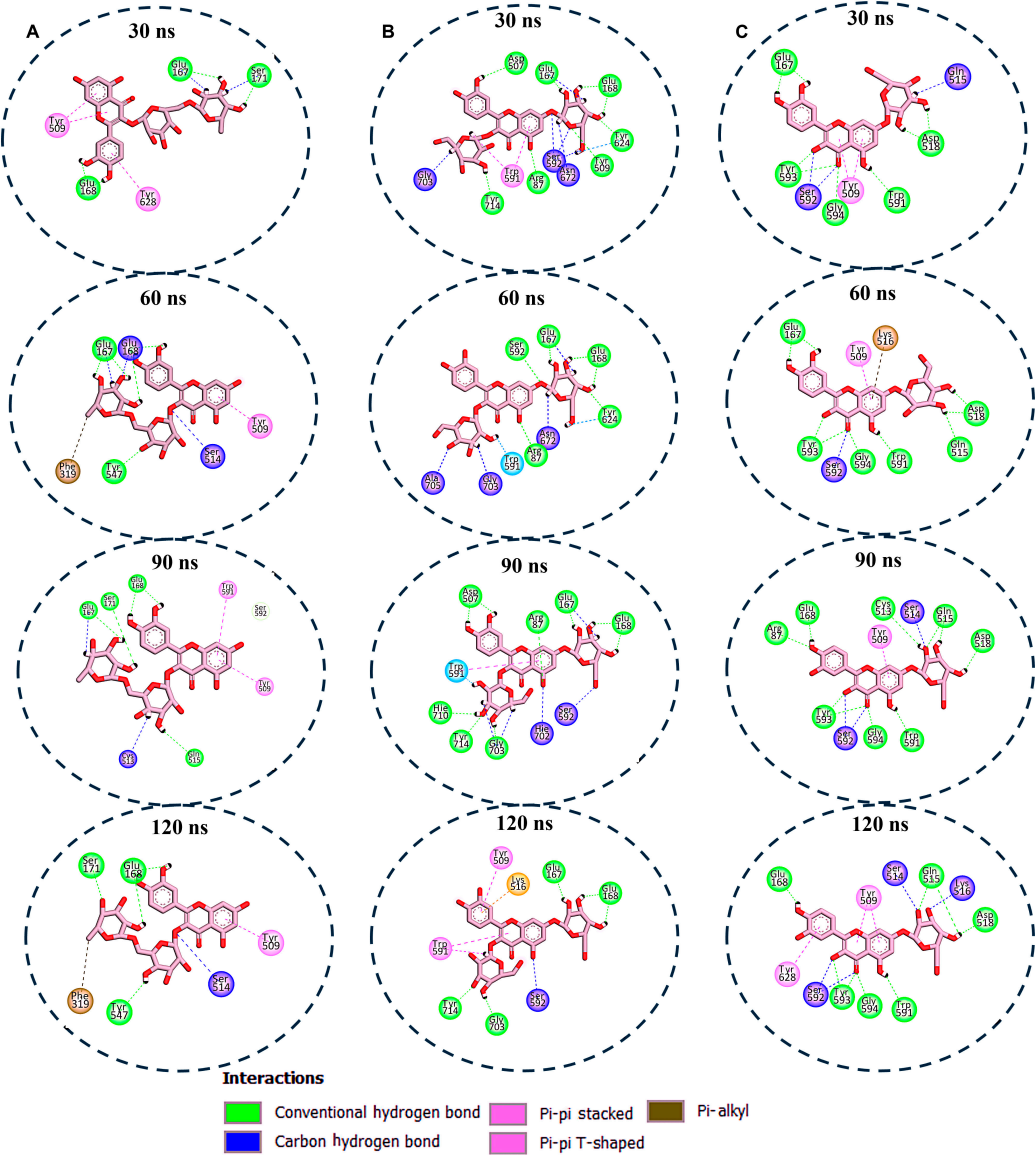
Enzyme–ligand interaction analysis over 120-ns MD simulation for (A) DPP4–rutin complex system showing stable interaction between the compounds and critical active site amino acid residues, indicating favorable binding. (B) The DPP4–Q37DG complex system demonstrated the most extensive binding with active site amino acid residues, including the catalytic residues Glu^167^, Glu^168^, Trp^591^, and Tyr^509^, which could inform its observed high binding free energy. (C) The DPP4–Q7G complex system also showed good binding with a substantial number of active site amino acid residues, which could also be responsible for its high binding free energy value.

**Fig. 8.**
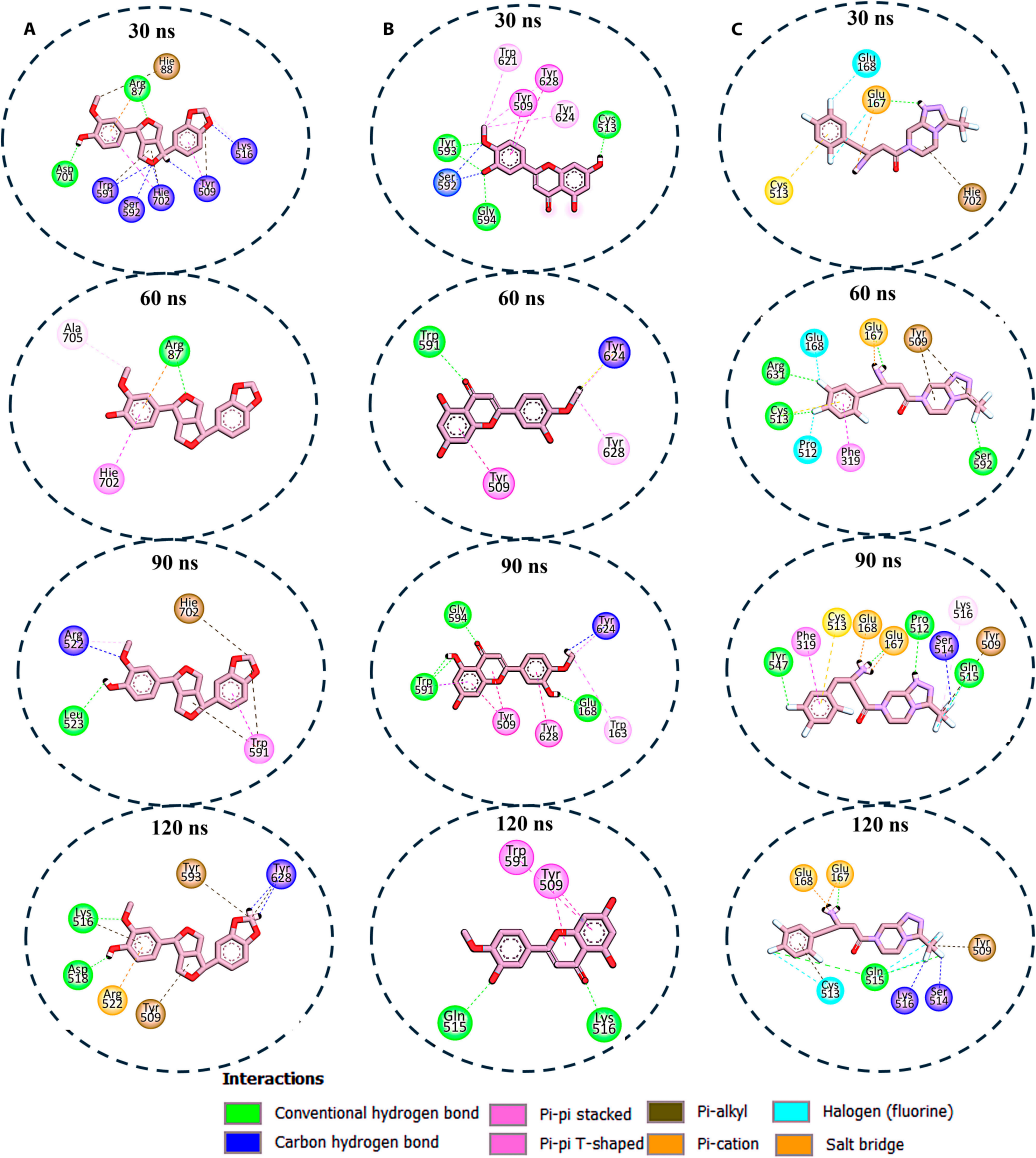
Enzyme–ligand interaction analysis over 120-ns MD simulation for the (A) DPP4–piperitol complex system, which showed that piperitol interacted with a moderate number of active site residues ranging from 6 to 8 amino acid residues throughout the simulation period. (B) The DPP4–diosmetin complex system showed a staggering form of binding interaction with active site residues fluctuating from 8 to 4, then 7, and stabilizing with 4 amino acid residues at the climax of 120-ns MD simulation. (C) Compared to the other 2 compounds, the DPP4–sitagliptin complex system shows progressive binding interaction, starting with 4 to 10 amino acid residues at roughly 90 ns and stabilizing with 7 amino acid residues at the end of the 120-ns MD period. Notably, it interacted with a unique halogen bond that provides binding specificity.

The interaction plots for the DPP4 system also showed that rutin maintained interaction with 5 to 7 amino acid residues, primarily engaging Glu^167^, Glu^168^, Tyr^509^, and Ser^171^ (Fig. 7A). Q37DG exhibited the most extensive binding network, maintaining 8 to 16 interactions and primarily engaging with the catalytic residues Glu^167^, Glu^168^, Trp^591^, and Tyr^509^ throughout the simulation (Fig. 7B). These interactions could be responsible for the most favorable binding energy reported for this compound in Table 1. Q7G showed robust engagement with key residues Asp^518^, Glu^167^, Trp^591^, and Tyr^509^, progressing from 13 to 14 interactions (Fig. 7C). The other compounds (diosmetin and piperitol) showed fluctuating interaction patterns (Fig. 8A and B), which accounted for their less favorable binding free energy (Table 1). The DPP4 reference compound sitagliptin demonstrated progressive interaction enhancement from 6 to 11 interactions, incorporating unique halogen bonds (Fig. 8C).

### Binding free energy decomposition analysis

**Fig. 9.**
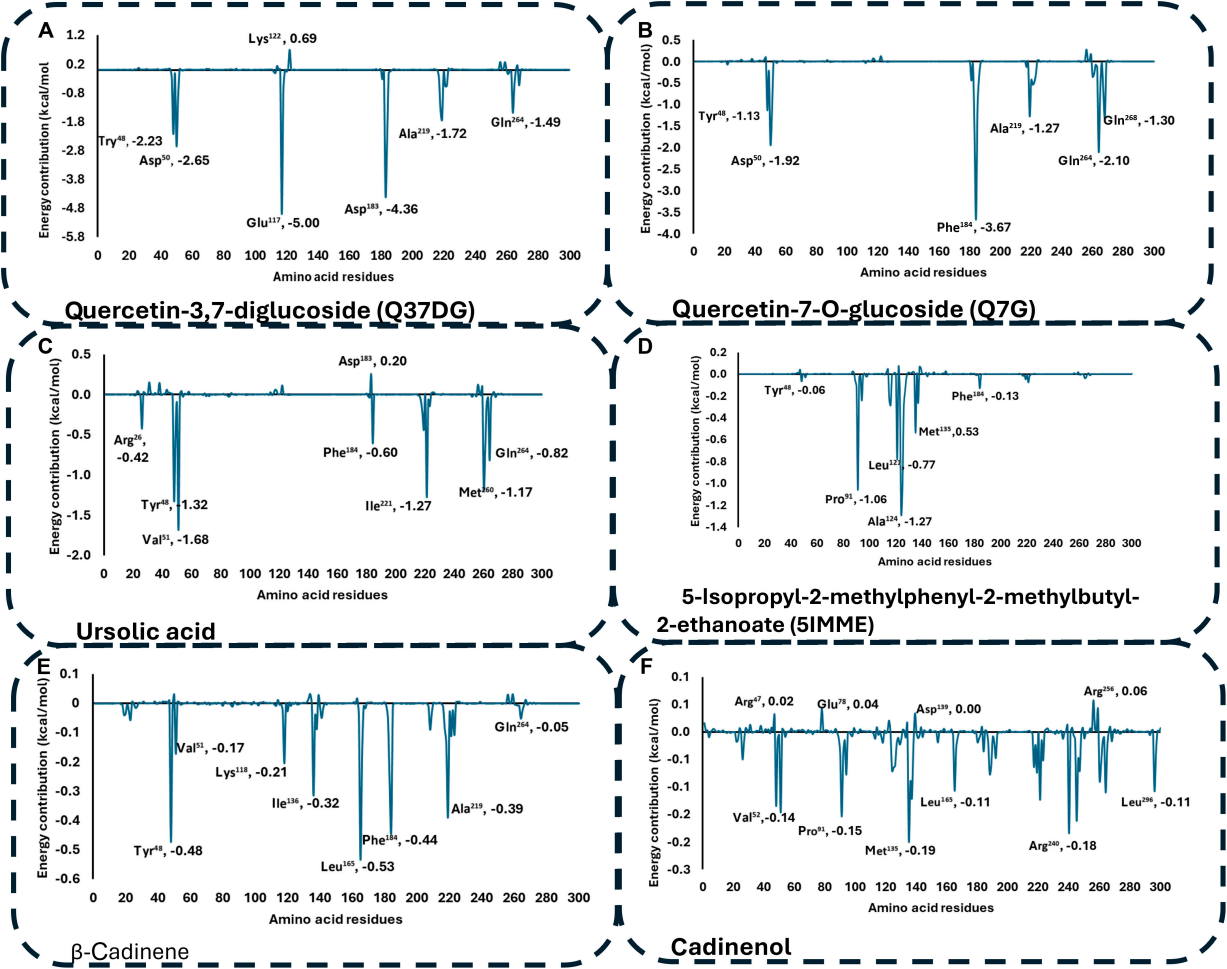
Per-residue energy decomposition analysis for the (A) PTP1B–Q37DG complex system with the most favorable energy profile when compared to other compounds interacting with catalytic amino acid residues Glu^117^ (−5.00 kcal/mol), Asp^183^ (−4.36 kcal/mol), Tyr^48^ (−2.20 kcal/mol), and Asp^50^ (−2.65 kcal/mol). (B) The PTP1B–Q7G complex system with the second most significant energy contributions from residues Phe^184^ (−3.67 kcal/mol), Asp^50^ (−1.92 kcal/mol), and Tyr^48^ (−1.13 kcal/mol). (C) The PTP1B–ursolic complex system showed the third most significant contributions from Val^51^ (−1.68 kcal/mol), Tyr^48^ (−0.48 kcal/mol), and Phe^184^ (−0.44 kcal/mol). (D) The next complex system is PTP1B–5IMME interacting with Ala^124^ (−1.27 kcal/mol) and Pro^91^ (−1.06 kcal/mol). (E and F) The energy contribution from the amino acid residues interacting with the PTP1B–cadinene and PTP1B–cadinenol systems was less than 1 kcal/mol, indicating low binding affinity and potentially limited antidiabetic effect.

To identify amino acid residues that make significant contributions to the total binding free energy, per-residue free energy decomposition was conducted. For the PTP1B system (PDB ID: 5t19), the PTP1B–Q37DG complex exhibited the most favorable interactions with catalytic residues Glu^117^ (−5.00 kcal/mol) and Asp^183^ (−4.36 kcal/mol) (Fig. 9A). This suggests that the compound competes for the catalytic site, supporting the high binding affinity recorded [46]. The PTP1B–Q7G complex followed, showing significant contributions from Phe^184^ (−3.67 kcal/mol) (Fig. 9B).

**Fig. 10.**
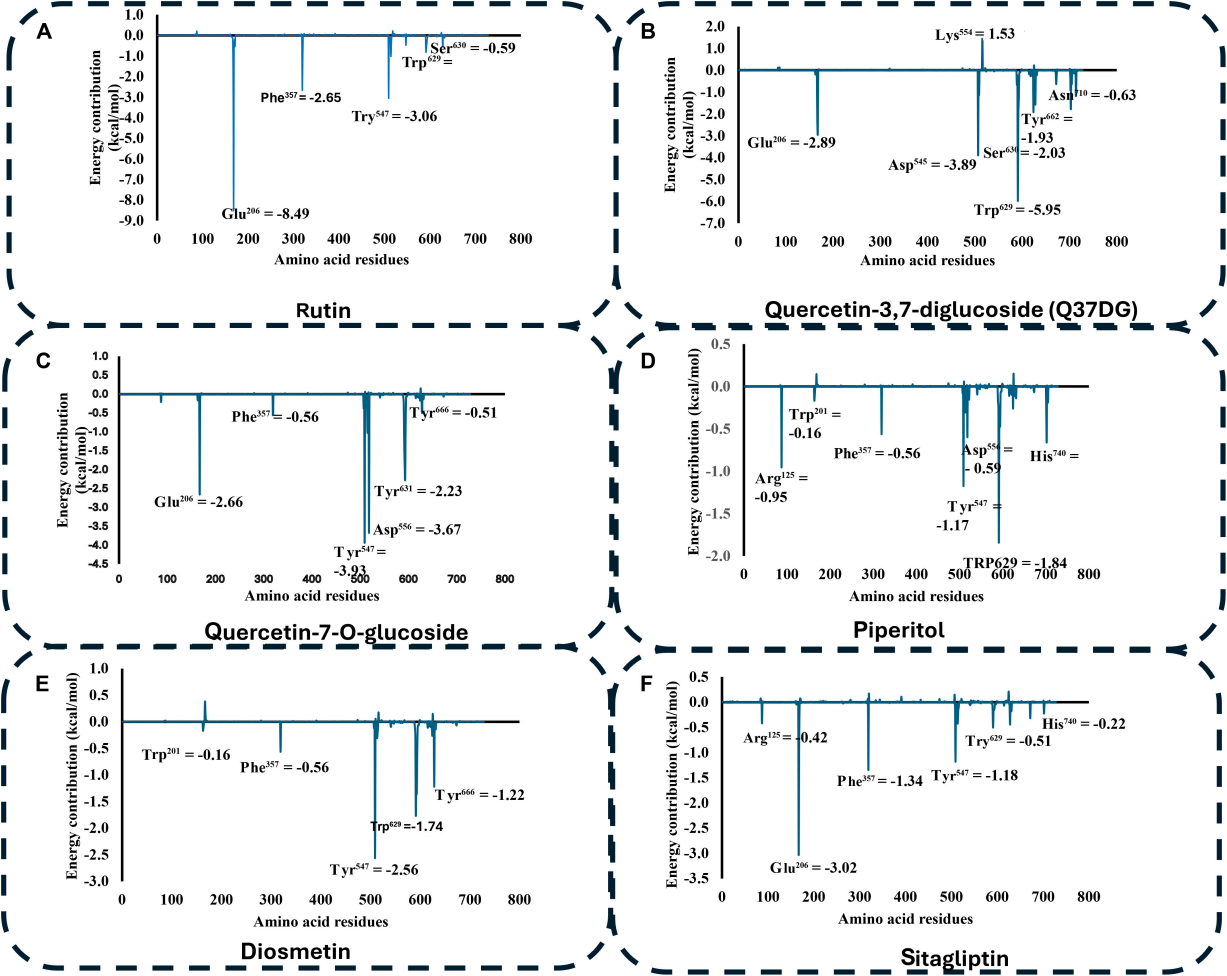
Per-residue energy decomposition analysis for the (A) DPP4–rutin complex system having the highest single-residue interaction with Glu^206^ (−8.49 kcal/mol). (B) The DPP4–Q37DG system has the second strongest single-residue interaction with Trp^629^ (−5.95 kcal/mol). (C) DPP4–Q7G complex system with robust binding through Asp^556^ (−3.67 kcal/mol). (D) The DPP4–piperitol complex system had limited favorable interactions with modest contributions from Trp^201^ (−0.16 kcal/mol). (E) The DPP4–diosmetin complex interacted with Tyr^547^ (−2.56 kcal/mol) and Trp^629^ (−1.74 kcal/mol). (F) The DPP4–sitagliptin complex showed relatively mild binding through Glu^206^ (−3.02 kcal/mol), Phe^357^ (−1.34 kcal/mol), and Tyr^547^ (−1.18 kcal/mol).

For the DPP4 system (PDB ID: 5t4e), the DPP4–rutin complex showed the most favorable interaction with residue Glu^206^ (−8.49 kcal/mol), a key component of the S2 subsite [34]. Other significant contributors included S1/S2 subsite residues Tyr^547^ (−3.06 kcal/mol) and the catalytic nucleophile Ser^630^ (−0.59 kcal/mol) (Fig. 10A). The DPP4–Q37DG complex ranked second, with high energy contributions from Trp^629^ (−5.95 kcal/mol) and catalytic residues Ser^630^ (−2.03 kcal/mol) and Glu^206^ (−2.89 kcal/mol). This observation showed that rutin has dual S1/S2 subsite occupancy and competitive inhibition via proteolytic blockade [28]. The ranking of relative binding stability based on multi-site engagement was Q37DG > rutin > Q7G > sitagliptin, suggesting that compounds could be promising candidates for diabetes treatment.

### Frontier molecular orbital properties

Correlation analysis between DFT-calculated frontier molecular orbital properties and binding free energies (Δ*G*_bind_) revealed significant structure–activity relationships for both target enzymes (Table 4). For PTP1B, molecular softness (ξ) exhibited the strongest negative correlation (*r* = −0.89), while energy gap (ΔE) showed the highest positive correlation (*r* = 0.88) (lower part of the diagonal of the correlation matrix). The softness-binding relationship demonstrates that Q37DG (ξ = 0.48 eV, Δ*G*_bind_ = −52.19 kcal/mol) achieves superior binding compared to β-cadinene (ξ = 0.29 eV, Δ*G*_bind_ = −11.14 kcal/mol) due to enhanced electronic reorganization capacity and polarizability, facilitating stronger van der Waals and π–π stacking interactions. Generally, molecular softness refers to a molecule’s ability to undergo electronic reorganization and polarization when interacting with other species. Softness has a significant implication for protein–ligand interactions because softness is the reciprocal of hardness (which is the difference between IP and EA). Therefore, soft molecules have low IP and high EA [47]. Increased polarizability in soft molecules enhanced their participation in van der Waals interactions and other hydrophobic interactions, such as π–π stacking, common aromatic systems in soft molecules [48]

**Table 4.** Compound frontier molecular orbital properties (eV) and binding free energy [Δ*G*_bind_ (kcal/mol)]

Compounds	Δ*G*_bind_	HOMO	LUMO	ΔE	IP	EA	η	ξ	χ	μ	ω
PTP1B											
Q37DG	−52.19	−5.80	−1.66	4.14	5.8	1.66	2.07	0.48	3.73	−3.73	3.36
β-Cadinene	−11.14	−6.09	0.88	6.97	6.09	−0.88	3.49	0.29	2.61	−2.61	0.97
Q7G	−38.63	−5.68	−1.55	4.13	5.68	1.55	2.07	0.48	3.62	−3.62	3.16
Cadinenol	−8.94	−6.16	0.80	6.96	6.16	−0.80	3.48	0.29	2.68	−2.68	1.03
5IMME	−14.93	−6.22	−0.91	5.31	6.22	0.91	2.66	0.38	3.57	−3.57	2.39
Ursolic acid	−23.95	−6.06	0.21	6.27	6.06	−0.21	3.14	0.32	2.93	−2.93	1.36
DPP4											
Rutin	−49.36	−5.64	−1.66	3.98	5.64	1.66	1.99	0.5	3.65	−3.65	1.31
Q37DG	−58.23	−5.68	−1.55	4.13	5.68	1.55	2.07	0.48	3.62	−3.62	3.16
Q7G	−39.95	−5.84	−1.49	4.35	5.84	1.49	2.18	0.46	3.67	−3.67	3.09
Piperitol	−20.07	−6.28	0.38	6.66	6.28	−0.38	3.33	0.3	2.95	−2.95	1.31
Diosmetin	−23.11	−5.51	−0.06	5.45	5.51	0.06	2.73	0.37	2.79	−2.79	1.42
Sitagliptin	−24.10	−6.33	−0.63	5.70	6.33	0.63	2.85	2.85	0.35	−3.48	2.12
Correlation matrix											
Parameters	Δ*G*_bind_	HOMO	LUMO	ΔE	IP	EA	η	ξ	χ	μ	ω
Δ*G*_bind_	1.00	−0.51	0.93	0.91	0.51	−0.93	0.91	−0.92	−0.90	0.90	−0.55
HOMO	−0.87	1.00	−0.57	−0.73	−1.00	0.57	−0.72	0.68	0.31	−0.31	0.10
LUMO	0.83	−0.69	1.00	0.98	0.57	−1.00	0.98	−0.99	−0.96	0.96	−0.52
ΔE	0.88	−0.77	0.99	1.00	0.73	−0.98	1.00	−1.00	−0.88	0.88	−0.46
IP	0.87	−1.00	0.69	0.77	1.00	−0.57	0.72	−0.68	−0.31	0.31	−0.10
EA	−0.83	0.69	−1.00	−0.99	−0.57	1.00	−0.98	0.99	0.96	−0.96	0.52
η	0.88	−0.77	0.99	1.00	0.72	−0.98	1.00	−1.00	−0.88	0.88	−0.46
ξ	−0.89	0.82	−0.98	−0.99	−0.68	0.99	−1.00	1.00	0.91	−0.91	0.44
χ	−0.76	0.58	−0.99	−0.96	−0.31	0.96	−0.88	0.93	1.00	−1.00	0.57
μ	0.76	−0.58	0.99	0.96	0.31	−0.96	0.88	−0.93	−1.00	1.00	−0.57
ω	−0.86	0.74	−0.99	−0.99	−0.10	0.52	−0.46	0.99	0.97	−0.97	1.00

I, ionization energy; EA, electron affinity; η, hardness; ξ, softness; μ, chemical potential; χ, electronegativity index; ω, electrophilicity index; Q37DG, quercetin-3,7-diglucoside; Q7G, quercetion-7-O-glucoside; 5IMME, 5-isopropy-2-methylphenyl-2-methylbutyl-2-ethanoate

For DPP4, EA displayed the strongest negative correlation (*r* = −0.93), while LUMO energy showed the highest positive correlation (*r* = 0.93) (upper part of the diagonal of the correlation matrix in Table 4). This indicates that compounds with lower LUMO energies have enhanced electrophilicity and electron-accepting capacity, which result in stronger protein interactions.

**Fig. 11.**
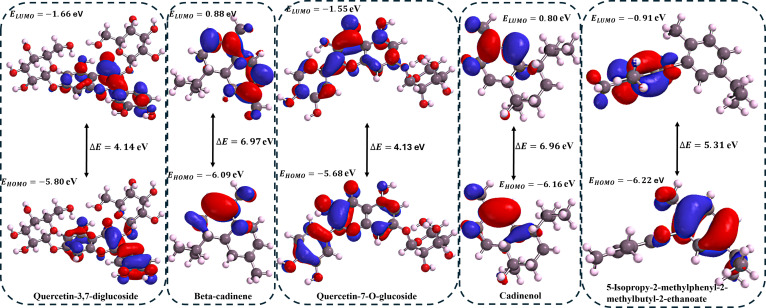
The DFT-derived frontier molecular properties showing Q37DG (Δ*E* = 4.14 eV) and Q7G (Δ*E* = 4.13 eV) with relatively smaller HOMO–LUMO gap and well-distributed frontier orbitals across the extended aromatic system.

**Fig. 12.**
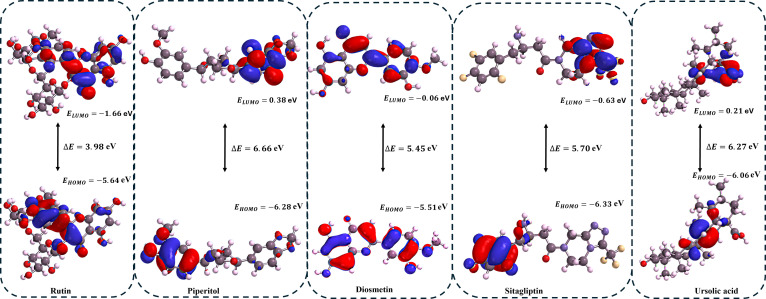
The DFT-derived frontier molecular properties showing rutin (Δ*E* = 3.98 eV) with a relatively smaller HOMO–LUMO gap and well-distributed frontier orbitals across the extended aromatic system. This explains its relatively higher binding energy and affinity for both PTP1B and DPP4 when compared to the other compounds (piperitol, diosmetin, sitagliptin, and ursolic acid).

This correlation explains the binding hierarchy: Q37DG (LUMO = −1.55 eV, Δ*G*_bind_ = −58.23 kcal/mol) > Q7G (LUMO = −1.49 eV, Δ*G*_bind_ = −39.95 kcal/mol) > sitagliptin (LUMO = −0.63 eV, Δ*G*_bind_ = −24.10 kcal/mol) > piperitol (LUMO = 0.38 eV, Δ*G*_bind_ = −20.07 kcal/mol). The DFT parameters successfully predict binding affinity trends, with quercetin derivatives (Q37DG, Q7G, and rutin) demonstrating superior reactivity through extended π-conjugation systems compared to saturated terpenes (β-cadinene, cardinenol, and 5IMME) with large HOMO–LUMO gaps and limited electronic delocalization. Generally, LUMO represents the lowest energy molecular orbital that contains no electrons in a molecule’s ground state. Lower LUMO energy is equivalent to higher electrophilicity of the molecule and implies a greater tendency of the molecule to accept electrons. This affects how the compound interacts with the electron-rich region of proteins. Orbital overlaps between the ligand’s LUMO and protein’s electron-rich regions (like aromatic amino acids) enhance binding. Frontier molecular orbital energy plots for the compounds are presented in Figs. 11 and 12.

### Pharmacokinetic and drug likeness prediction

The pharmacokinetic and drug-likeness profiles of the prioritized compounds were evaluated using Swiss-ADME to assess their potential for clinical translation. All compounds demonstrated bioavailability scores between 17% and 55% and exhibited good water solubility [22] (Table 5). Notably, the lead flavonoids Q37DG, Q7G, and rutin showed no CYP450 inhibition, suggesting a favorable safety profile regarding drug–drug interactions [19].

**Table 5.** Pharmacokinetic and drug likeness prediction for studied compounds using Swiss ADME

Molecule	Diosmetin	Q37DG	Q7G	Rutin	Piperitol	Cadinene	Cadinenol	5IMME
No. H-bond donor	3	11	8	10	1	0	0	1
No. H-bond acceptor	6	17	12	16	6	0	1	2
Lipophilicity	2.47	0.79	0.84	0.46	3.25	3.36	3.15	3.36
Bioavailability score	0.55	0.17	0.17	0.17	0.55	0.55	0.55	0.55
Synthetic accessibility	3.05	6.60	5.31	6.52	4.19	4.35	4.29	2.35
No. of Lipinski violations	0	3	2	3	0	1.00	0	0
No. of Veber violations	0	1	1	1	0	0	0	0
No. of Egan violations	0	1	1	1	0	0	0	0
Water solubility	M-soluble	Soluble	Soluble	Soluble	Soluble	Soluble	Soluble	M-soluble
GI absorption	High	Low	Low	Low	High	Low	High	High
BBB permeability	No	No	No	No	Yes	No	Yes	Yes
Pgp substrate	No	No	No	Yes	No	No	No	No
CYP1A2 inhibitor	Yes	No	No	No	No	No	No	No
CYP2C19 inhibitor	No	No	No	No	No	Yes	No	Yes
CYP2C9 inhibitor	Yes	No	No	No	No	Yes	No	No
CYP2D6 inhibitor	Yes	No	No	No	Yes	No	No	No
CYP3A4 inhibitor	Yes	No	No	No	Yes	No	No	No

GI, gastrointestinal; BBB, blood–brain barrier; Pgp, P-glycoprotein; CYP, cytochrome P450; Q7G, quercetin-7-O-glucoside; Q37DG, quercetin-3,7-diglucoside; 5IMME, 5-isopropyl-2-methylpheneyl-2-methylbutyl-2-ethanone; M-soluble, moderately soluble

However, a candid assessment of their developability reveals significant pharmacokinetic limitations. Despite their high theoretical affinity for PTP1B and DPP4, Q37DG, Q7G, and rutin exhibit several drug-likeness violations, including high molecular weight (exceeding 600 Da for Q37DG and rutin) and high polarity, which contribute to the Lipinski, Veber, and Egan violations observed (Table 5). These features result in poor predicted GI absorption, representing a major “translation bottleneck” from computational models to clinical reality. Consequently, these compounds should be categorized as early-stage pharmacological probes or structural templates rather than near-drug candidates.

To overcome these absorption barriers, several therapeutic optimization pathways are proposed. First, the therapeutic potential of these glycosides may rely on in vivo deglycosylation. Intestinal microbiota-mediated deglycosylation likely releases the more lipophilic aglycone (quercetin), which may exhibit superior absorption and systemic activity. Furthermore, future research should focus on structural optimization to reduce molecular weight or the development of advanced delivery systems. Strategies such as nanoparticle encapsulation, nano-emulsions, or phospholipid complexes could significantly enhance the bioavailability of these bulky polar molecules. Additionally, prodrug designs intended to temporarily mask polar glycoside moieties may improve membrane permeability, allowing these leads to realize their full therapeutic potential.

### PTP1B and DPP4 inhibitory activity of the essential oils: A dose–response analysis

**Fig. 13.**
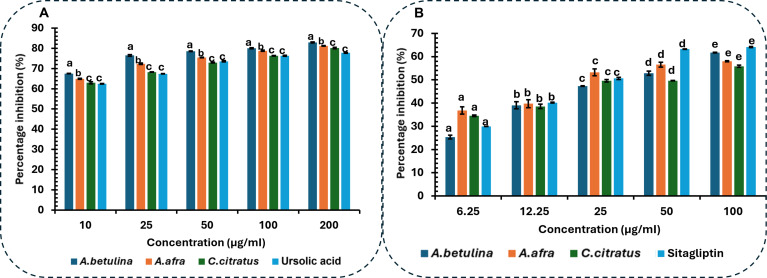
Inhibitory effect of selected essential oils on key metabolic enzymes associated with type 2 diabetes: (A) protein tyrosine phosphatase 1B (PTP1B) with inhibitory activity compared against ursolic acid as positive control $\begin{array}{c} \left(P=7.38\times 10^{-10};F_{\text{crit}}=3.25916\right) \end{array}$ and (B) dipeptidyl peptidase 4 (DPP4) with inhibitory activity compared against sitagliptin as positive control $\begin{array}{c} \left(P=8.61\times 10^{-7};F_{\text{crit}}=3.25917\right) \end{array}$. Results are presented as the mean ± standard deviation (SD) of triplicate determinations (*n* = 3). Different letters (a to e) indicate significant differences between essential oils at the same concentration (*P* < 0.05), determined by two-way analysis of variance (ANOVA) followed by Tukey’s honestly significant difference (HSD) test (HSD = 2.0352 for PTP1B and HSD = 6.999 for DPP4).

**Fig. 14.**
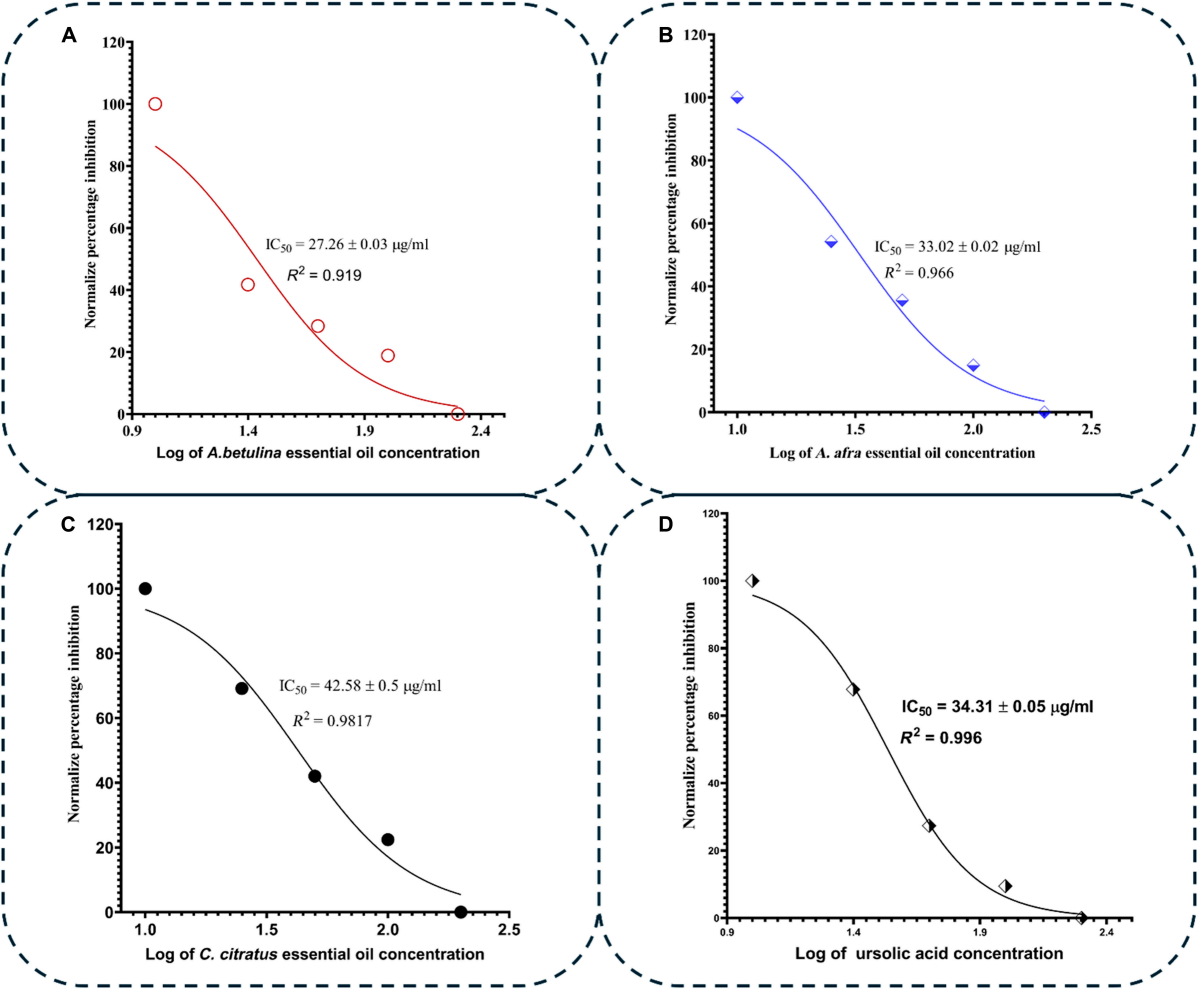
Normalized percentage inhibition against the logarithm of the essential oil concentration for PTP1B IC_50_ determination for (A) *A. betulina*, (B) *A. afra*, and (C) *C. citratus* essential oils and (D) ursolic acid.

The inhibitory capabilities of *A. betulina*, *A. afra*, and *C. citratus* essential oils, as presented in Fig. 13, showed that all tested oils exhibited positive inhibition against PTP1B and DPP4. The inhibitory percentage ranged from 77.77 ± 0.11% to 82.92 ± 0.13% at the highest test concentration and from 62.46 ± 0.21% to 67.52 ± 0.30% at the lowest concentration for PTP1B. At the peak screened concentration (200 μg/ml), all essential oils exhibited significantly (*P* < 0.05) higher PTP1B inhibition than the control, ursolic acid (77.77 ± 0.11%), except for *C. citratus* essential oil (Fig. 13A; $P=7.38\times 10^{-10},F_{\text{crit}}=3.259$). Notably, *A. betulina* essential oil had the highest average inhibitory percentage at this concentration (82.91 ± 0.13%) and demonstrated the greatest inhibition, with an IC_50_ of 27.26 ± 0.03 μg/ml. The IC_50_ values of the screened essential oils, which varied in their effectiveness at inhibiting PTP1B, are presented in Fig. 14. *A. betulina* and *A. afra* essential oils showed greater inhibitory effectiveness, as evidenced by lower IC_50_ values relative to that of the control, ursolic acid (34.31 ± 0.05 μg/ml), suggesting their potential potency at inhibiting PTP1B and probable utility for diabetes treatment.

**Fig. 15.**
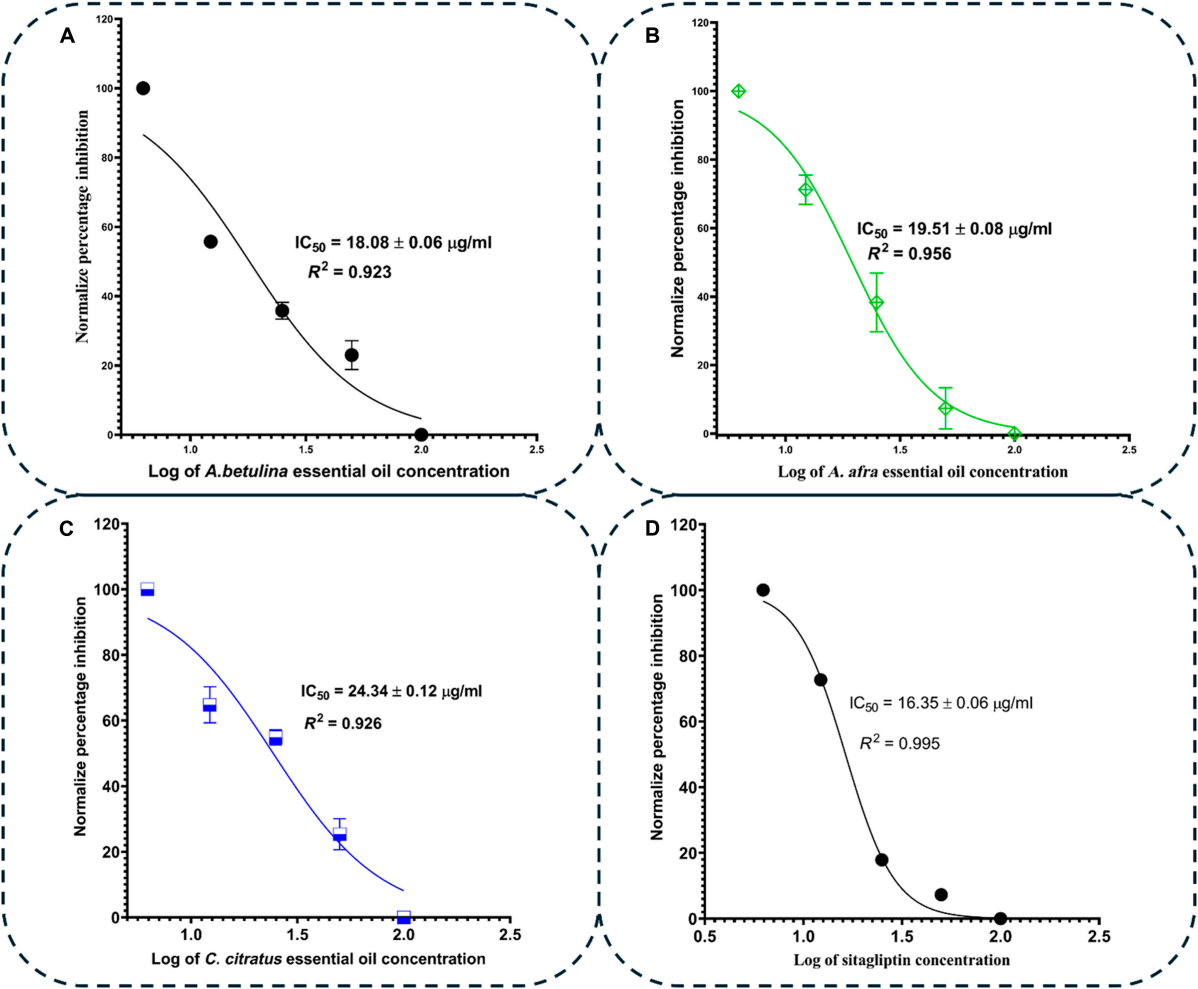
Normalized percentage inhibition against the logarithm of the essential oil concentration for DPP4 IC_50_ determination for (A) *A. betulina*, (B) *A. afra*, and (C) *C. citratus* essential oils and (D) sitagliptin.

Similarly, the inhibitory percentage for DPP4 ranged from 55.83 ± 0.49% to 64.09 ± 0.24% at the highest test concentration and from 25.35 ± 0.80% to 44.53 ± 0.36% at the lowest concentration $\left(P=8.61\times 10^{-7},F_{\text{crit}}=3.259\right)$.At the peak screened concentration $\left(100\;\mathrm{\mu g}/\mathrm{ml}\right)$, the control, sitagliptin exhibited higher DPP4 percentage inhibition compared to all the essential oils, although this difference was not statistically significant P<0.05 (Fig. 13B). The DPP4 IC_50_ values of the screened essential oils are presented in Fig. 15, which indicates a comparable value to the control, sitagliptin (16.35 ± 0.06 μg/ml)—suggesting their potency at inhibiting DPP4 and their potential for diabetes treatment. Both the PTP1B and DPP4 assays revealed that *A. betulina* essential oil demonstrated superior inhibitory potency when compared to the other 2 oils. The findings suggest that the total phytochemical matrix of *A. betulina* contains multiple high potency leads, aligning with literature reports on the experimental antidiabetic properties of *C. citratus* constituents [49].

### Predicted PTP1B and DPP4 inhibitory activity of identified lead compounds

**Fig. 16.**
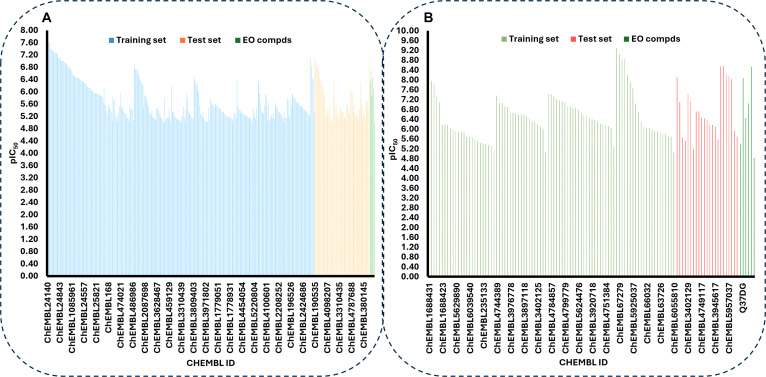
Distribution of the inhibitory activity of the training and test set for (A) PTP1B and (B) DPP4 dataset.

To explore the broader pharmacological potential of the *A. betulina* metabolome beyond the volatile fraction, the inhibitory potential of the prioritized lead compounds (Q37DG, Q7G, and rutin) was evaluated using GA-QSAR modeling. This computational exercise was designed to identify high-affinity molecular scaffolds within the plant’s nonvolatile chemical space, independent of the essential oil bioassays. The distribution of the inhibitory activity of 398 (PTP1B) and 114 (DPP4) ChEMBL compounds used for the construction of the models in this study is presented in Fig. 16, which shows that the activity values of compounds in the text set are within the activity range of the training set. The genetic GA-QSAR models obtained from these datasets are presented in Eqs. (8) (PTP1B) and (9) (DPP4).

$$\begin{array}{c} {\mathrm{pIC}}_{50}=6.697\left(\pm 0.111\right)+0.927\left(\pm 0.070\right)\;R\_\text{TpiPCTPC}\\ -2.007\left(\pm 0.177\right)\;\text{nRing}+0.743\left(\pm 0.146\right)\;\text{ATSC}4m\\ -0.767\left(\pm 0.086\right)\;\text{GATS}3p+3.407\left(\pm 0.188\right)\;\text{fragC}\\ -0.267\left(\pm 0.105\right)\;\text{MATS}7e-1.308\left(\pm 0.118\right)\;\text{MAXDP}-\\ 0.756\left(\pm 0.089\right)\;\text{AATS}3e \end{array}$$ (8)

$$\begin{array}{c} {\mathrm{pIC}}_{50}=-5.494\left(\pm 0.816\right)-2.521\left(\pm 0.952\right)\;\text{SIC0}+2.015\\ \left(\pm 0.174\right)\;\text{GATS}7m+6.098\left(\pm 0.256\right)\;\text{SpMax}1\_\mathrm{Bhv}\\ +3.804\left(\pm 0.428\right)\;\mathrm{JGT}+0.044\left(\pm 0.003\right)\;\text{apol}-9.424\\ \left(\pm 0.382\right)\;\text{SpMin}2\_\mathrm{Bhv}+0.119\left(\pm 0.010\right)\;\text{ATSC}6p\\ +10.345\left(\pm 0.592\right)\;\text{AATSC}8p \end{array}$$ (9)

**Fig. 17.**
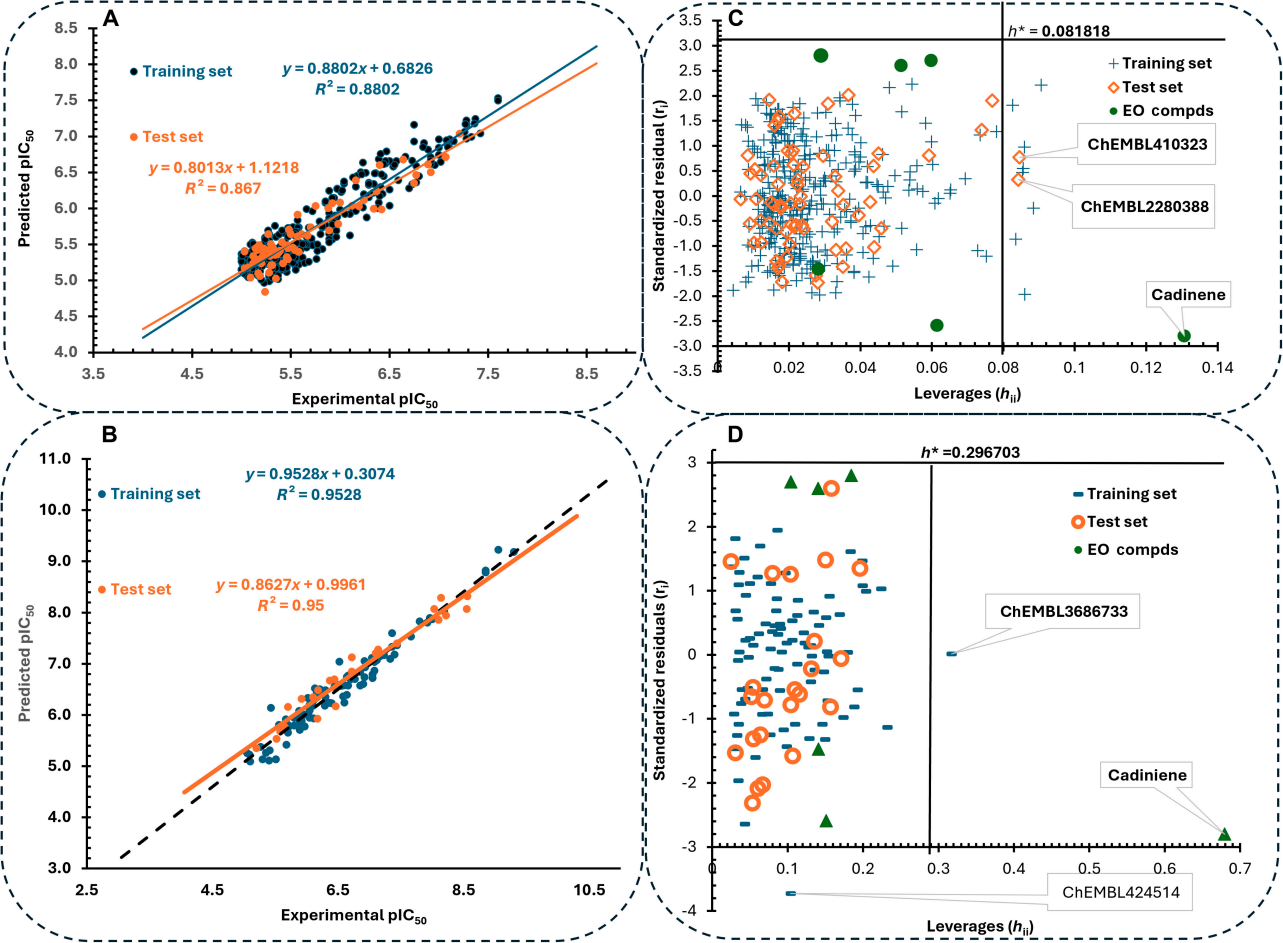
Validation and applicability domain (AD) analysis of the developed QSAR models. (A and B) Correlation plots of experimental versus predicted pIC_50_ values for the training (blue) and test (orange) sets, demonstrating the internal and external predictive power of the models. (C and D) Williams plots (standardized residuals versus leverages) illustrating the applicability domain of the models; the vertical line represents the warning leverage (h*), and the horizontal lines at ±3 represent the outlier thresholds. Compounds such as cadinene and specific ChEMBL entries are identified as influential points or outliers within the chemical space.

The alphanumerical components of the right hand side of Eqs. (8) and (9) are the molecular descriptors defined in Table 6. The validation parameters for the models presented in Table 7 indicate that the models are robust and have good predictive ability for both training and test set data. Hence, the models are suitable for predicting the activity of compounds not included in the training set, provided the compounds are within the AD of the models. Figure 17A and B shows a linear relationship between the predicted activity of both training and test set compounds and the experimental values, with coefficients of determination *R*^2^ > 0.7 for the PTP1B and DPP4 datasets, respectively. This linear relationship further strengthens the predictive reliability of the model. After establishing the predictive ability of the model, the molecular descriptors contained in the models were calculated for the lead compounds using the PaDEL descriptor software, prepared, and imputed into the model to evaluate the hypothetical inhibitory potency for the lead compound IC_50_ (predicted) (μg/ml) (Table 8).

**Table 6.** Description of descriptors contained in the models

Sr. no.	Symbols	Contribution	Description
PTP1B QSAR model [Eq. (8)]			
1	R_TpiPCTPC	Positive	Reduced tripeptide position-specific tripeptide composition
2	nRing	Negative	Number of rings
3	ATSC4m	Positive	Centered Broto–Moreau autocorrelation of lag 4 weighted by mass
4	GATS3p	Negative	Geary autocorrelation of lag 3 weighted by polarizability
5	fragC	Positive	Complexity of a system
6	MATS7e	Negative	Moran autocorrelation of lag 7 weighted by Sanderson electronegativity
7	MAXDP	Negative	Maximal electrotopological positive variation
8	AATS3e	Negative	Average autocorrelation topological structure of Lag 3 weighted by Sanderson electronegativity
DPP4 QSAR model [Eq. (9)]			
1	SIC0	Negative	Structural information content index (neighborhood symmetry of 0-order)
2	GATS7m	Positive	Geary autocorrelation of lag 7 weighted by mass
3	SpMax1_Bhv	Positive	The largest eigenvalue of a modified Burden adjacency matrix, weighted by atomic van der Waals volumes
4	JGT	Positive	Global topological charge index
5	apol	Positive	Sum of the atomic polarizabilities (including implicit hydrogens)
6	SpMin2_Bhv	Negative	Second smallest eigenvalue of the modified Burden adjacency matrix weighted by atomic van der Waals volumes
7	ATSC6p	Positive	Centered Broto–Moreau autocorrelation of lag 6 weighted by polarizability
8	AATSC8p	Positive	Average autocorrelation (centered) - Lag 8 - Atomic polarizabilities

**Table 7.** Validation parameters for both PTP1B and DPP4 QSAR models

Parameters	Values		Threshold	Comment	Reference
	PTP1B QSAR model	DPP4 QSAR model			
Internal validation					
SEE	0.219	0.202			
r^2	0.880	0.952	>0.6	Passed	[50]
r^2 adjusted	0.877	0.948	>0.6	Passed	[50]
PRESS	15.409	206.871			
Leave-one-out (LOO) result					
Q^2^	0.873	0.942	>0.6	Passed	[50]
Average rm^2(LOO)	0.829	0.919	>0.6	Passed	[50]
Delta rm^2(LOO)	0.082	0.035			[50]
External validation parameters (without scaling)					
r^2	0.867	0.949	>0.6	Passed	[27]
r0^2	0.862	0.940	>0.6	Passed	[27]
reverse r0^2	0.814	0.923	>0.6	Passed	[27]
RMSEP	0.206	0.257			
Q^2^_f1_/R^2(Pred)	0.863	0.942	>0.6	Passed	[27]
Q^2^_f2_	0.862	0.938	>0.6	Passed	[27]
External validation parameters (after scaling)					
Average rm^2(test)	0.769	0.818	>0.6	Passed	[51]
Delta rm^2(test)	0.100	0.057	>0.6	Passed	[51]
Error-based judgement of test set predictions					
Mean absolute error (MAE; 95% data)	0.156	0.198			[52]
Standard deviation of absolute error (SD; 95% data)	0.102	0.112			[52]
Model quality based on MAE-based criteria	GOOD	GOOD			
Golbraikh and Tropsha acceptable model criteria					
Q^2	0.873	0.942	>0.5	Passed	[53]
r^2	0.867	0.949	>0.6	Passed	[53]
|r0^2-r’0^2|	0.047	0.017	<0.3	Passed	[53]
k	1.001	0.992	0.85 < k < 1.15	Passed	[53]
[(r^2-r0^2)/r^2]	0.006	0.001	(r^2 − r0^2)/r^2) < 0.1	Passed	[53]
k’	0.998	1.006	0.85 < k’ < 1.15	Passed	[53]
[(r^2 − r’0^2)/r^2]	0.061	0.028	(r^2 − r’0^2)/r^2) < 0.1	Passed	[53]

**Table 8.** Model descriptors and predicted IC_50_ values for the lead compounds

PTP1B QSAR model [Eq. (8)]												
Name	R_TpiPCTPC	nRing	ATSC4m	GATS3p	fragC	MATS7e	MAXDP	AATS3e	pIC_50_ (Pred.)	IC_50_ (mol/dm^3^)	MW (g/mol)	IC_50_ (μg/ml)
Diosmetin	7.249	3	24.31	1.084	114.06	−0.043	5.124	8.299	4.955	1.11E−05	300.26	3.33
Q37DG	3.939	5	−120.67	1.021	412.17	0.014	6.737	8.565	6.213	6.12E−07	626.50	0.38
Q7G	4.419	4	−135.32	1.071	240.12	0.086	5.654	8.513	5.531	2.95E−06	464.40	1.37
Rutin	3.673	5	−137.09	1.055	403.16	0.028	6.570	8.544	5.273	5.33E−06	610.50	3.26
Cadinene	1.994	2	−390.42	1.254	46.00	−0.364	0.500	7.141	5.978	1.05E−06	204.35	0.21
Piperitol	3.251	5	−495.29	1.212	250.06	−0.104	3.820	7.818	5.146	7.14E−06	356.40	2.55
DPP4 QSAR model [Eq. (9)]												
Name	SIC0	GATS7m	SpMax1_Bhv	JGT	apol	SpMin2_Bhv	ATSC6p	AATSC8p	pIC_50_ (Pred.)	IC_50_ (mol/dm^3^)	MW (g/mol)	IC_50_ (μg/ml)
Diosmetin	0.292	0.970	3.913	0.538	40.97	1.891	2.007	−0.046	5.398	4.00E−06	300.26	1.20
Q37DG	0.249	0.855	3.984	0.556	81.16	1.899	3.257	−0.003	8.081	8.30E−09	626.50	0.01
Q7G	0.268	0.824	3.935	0.561	60.59	1.911	1.380	−0.002	6.449	3.56E−07	464.40	0.17
Rutin	0.248	0.888	3.896	0.565	80.36	1.909	0.918	−0.021	7.046	9.00E−08	610.50	0.05
Cadinene	0.182	1.253	3.867	0.492	42.40	1.745	−0.405	0.109	8.540	2.88E−09	204.35	0.00
Piperitol	0.259	1.088	3.899	0.407	53.35	1.928	−2.051	−0.048	4.828	1.48E−05	356.40	5.29

Q37DG, quercetin-3,7-diglucoside; Q7G, quercetin-7-O-glucoside

The GA-QSAR models predicted high theoretical inhibitory potency for the flavonoid glycosides (Table 8). For PTP1B, predicted IC_50_ values were 0.38, 1.37, and 3.26 μg/ml for Q37DG, Q7G, and rutin, respectively. For DPP4, the models generated extraordinarily potent nanomolar predictions (0.01, 0.17, and 0.05 μM). While these values suggest high theoretical affinity for the target enzyme active sites, they must be interpreted strictly as theoretical scaffold prioritization outputs rather than absolute indicators of biological efficacy.

Crucially, these QSAR predictions do not explain or validate the in vitro activity observed for the essential oils, as the 2 represent chemically distinct fractions. Furthermore, the high theoretical potency of these bulky, polar glycosides is contrasted by their poor predicted GI absorption and pharmacokinetic liabilities identified in the ADME analysis. Therefore, these compounds are characterized as early-stage structural templates that require significant optimization or specialized delivery systems to overcome the identified translation bottlenecks. These findings serve to prioritize specific nonvolatile metabolites for future isolation and experimental validation, highlighting the plant’s broader potential as a source of diverse anti-diabetic leads. Williams plots [Fig. 17C (PTP1B) and D (DPP4)] confirmed that these compounds were within the model’s AD, making the predictions reliable benchmarks for future bioprospecting.

It is important to acknowledge the inherent limitations of this study, particularly the gap between the in vitro validation of the essential oils and the in silico modeling of the flavonoid glycosides. While the *A. betulina* essential oil demonstrated potent experimental inhibition of PTP1B and DPP4, this activity is likely driven by volatile terpenoids and synergistic matrix effects. The identified computational leads, Q37DG and Q7G, represent theoretical scaffolds from the plant’s broader nonvolatile metabolome rather than the active constituents of the tested oil. Due to the resource-intensive nature of isolating or synthesizing these specific glycosylated standards, they were not subjected to individual in vitro validation in this work. Consequently, these findings should be interpreted as a prioritized computational roadmap for future bioprospecting. Further research involving bioassay-guided fractionation of polar extracts is required to experimentally confirm the inhibitory potential of these specific flavonoids.

## Conclusion

This study successfully integrated structure-based modeling, MD simulations, in vitro enzyme inhibition assays, and GA-driven QSAR to identify potent antidiabetic leads from *A. betulina*, *A. afra*, and *C. citratus* constituents. The findings highlight *A. betulina* essential oil as the most effective source of dual-target inhibitors against PTP1B (IC₅₀ = 27.26 μg/ml) and DPP4 (IC₅₀ = 42.28 μg/ml). Computational analysis identified Q37DG and Q7G as the primary bioactive leads, demonstrating favorable energetic profiles within the target’s binding pocket relative to the standards, although experimental assays remain the definitive measure of potency. While the MMGBSA binding free energies were significantly negative (ranging from −52 to −58 kcal/mol), these were utilized as relative scores to rank the stability of the complexes. The binding free energy is largely driven by van der Waals interactions and strategic engagement with catalytic residues such as Glu^117^ and Asp^183^ in PTP1B, and Glu^206^ and Ser^630^ in DPP4. The GA-QSAR models further supported the finding, predicting high inhibitory potencies for the compounds. However, the identification of significant pharmacokinetic hurdles, including poor predicted GI absorption and high molecular weight, necessitates the classification of these flavonoids as early-stage pharmacological probes. The compounds identified could serve as vital structural templates for dual inhibition of PTP1B and DPP4. Future research must bridge the gap between computational affinity and clinical reality through structural optimization, metabolite-based activity studies, and advanced formulation strategies to enhance oral bioavailability. Furthermore, well-funded studies focusing on bioassay-guided isolation of the identified compounds to definitively correlate the theoretical binding affinities with experimental IC_50_ values are encouraged. Nevertheless, this work establishes a strong scientific basis for the traditional use of *A. betulina* while providing a roadmap for the development of high-efficacy, plant-derived antidiabetics.

## Data Availability

The data are contained within the article or the Supplementary Materials.
