# Supplementary material for: Dual PTP1B/DPP4 Inhibitory Potential of Agathosma betulina, Cymbopogon citratus, and Artemisia afra: Structure-Based Modeling of Phytochemical Leads and Essential Oil Bioassays
Source: Comput Struct Biotechnol J. 2026 Apr 16;35(1):0028. doi: 10.34133/csbj.0028 (PMC13084071; doi:10.34133/csbj.0028)
Supplement: Supplementary 1 — Tables S1 and S2 [file csbj.0028.f1.docx]

**List of supplementary tables**

Table S1. Docking scores of bioactive compounds in the essential oils (*A.betulina, A.afra* and *C.citratus*) scores against PTP1B and DPPIV

Table S2. Table S2. Correlation analysis between binding energy and other thermodynamic parameters

Table S1. Docking scores of bioactive compounds in the essential oils (*A.betulina, A.afra* and *C.citratus*) scores against PTP1B and DPPIV

|  | **PTP1B** |  | **DPPIV** |  |
| --- | --- | --- | --- | --- |
| Sr.No | Ligand | Docking score | Ligand | Docking score |
| 1 | Quercetin_3,7-diglucoside | -9 | Rutin | -9.6 |
| 2 | Beta-cadinene | -8.7 | Sitagliptin (reference compd.) | -9.4 |
| 3 | Cadin-4-en-10-ol | -8.5 | Quercetin_3,7-diglucoside | -9.2 |
| 4 | Quercetin_7-O-glucoside | -8.5 | Quercetin_7-O-glucoside | -9.2 |
| 5 | 5-isopropy-2-methylphenyl-2-methylbutyl-2-ethanoate | -8.4 | Piperitol | -8.5 |
| 6 | Alpha calacorene | -8.4 | 8,8'-Bi-p-cymene | -8.3 |
| 7 | Piperitol | -8.4 | Diosmetin | -8.3 |
| 8 | Trans-cadina-1(2),4-diene | -8.4 | Aromadendrene | -7.9 |
| 9 | Gamma-muurolene | -8.2 | 1-(Hydroxymethyl)-4-(4-metho-xyphenyl)-10-oxa-4-aza-tricyclo-[5-2-1-0-20-6]-dec-8-ene-3,_5-dione | -7.8 |
| 10 | Calamenene | -8.1 | 2-Isopropyl-5-methylphenyl-2-methylbut-2-enoate | -7.4 |
| 11 | Delta-cadinene | -8.1 | 5-isopropy-2-methylphenyl-2-methylbutyl-2-ethanoate | -7.4 |
| 12 | Delta-pinene | -8.1 | Calamenene | -7.4 |
| 13 | 4-Isopropyl-6-methyltetralone | -8 | Germacrene_D | -7.4 |
| 14 | Rutin | -8 | Phenol-2-(4-diethylaminophenyl-iminomethyl)-_ | -7.4 |
| 15 | Alpha-cadinol | -7.9 | Squalene | -7.3 |
| 16 | Cedrelanol | -7.9 | 3-methylbut-2-enoic acid,4-isopropylphenyl ester | -7.2 |
| 17 | Chamazulene | -7.9 | Chamazulene | -7.2 |
| 18 | Diosmetin | -7.9 | Alpha-acorenol | -7.1 |
| 19 | Alpha-cadinene | -7.8 | Alpha-bisabolo | -7.1 |
| 20 | Epi-a-muurolol | -7.8 | Alpha-muurolene | -7.1 |
| 21 | Epi-cubebol | -7.8 | Ar-curcumene | -7.1 |
| 22 | 2-Isopropyl-5-methylphenyl-2-methylbut-2-enoate | -7.7 | Gamma-selinene | -7.1 |
| 23 | Gamma-cadinene | -7.6 | Tau-muurolol | -7.1 |
| 24 | Cis-carvylacetate | -7.4 | Cubenol | -7 |
| 25 | Trans-carveyl acetate | -7.4 | Cuparene | -7 |
| 26 | 2H-pyran-2-carboxylic-acid-5-ethylidene-5-6 | -7.3 | Germacrene_A | -7 |
| 27 | 4-epi-cubedol | -7.3 | Myrtenyl acetate | -7 |
| 28 | Ar-curcumene | -7.3 | 2H-pyran-2-carboxylic-acid-5-ethylidene-5-6 | -6.9 |
| 29 | L-Menthyl_acetate | -7.3 | Alpha-farnesene | -6.9 |
| 30 | P-menth-1-en-9-yl-acetate | -7.3 | Benzyl-benzoate | -6.9 |
| 31 | 3-methylbut-2-enoic acid,4-isopropylphenyl ester | -7.2 | Beta-cadinene | -6.9 |
| 32 | 3-methylbut-2-enoic acid,4-nitrophenyl ester | -7.2 | Beta-farnesene | -6.9 |
| 33 | Cyclohexadecane | -7.2 | Delta-cadinene | -6.9 |
| 34 | Isocaryophyllene | -7.2 | Delta-pinene | -6.9 |
| 35 | Trifluoroacetyllavandulol | -7.2 | Geranyl-n-butyrate | -6.9 |
| 36 | Cuparene | -7.1 | Globulol | -6.9 |
| 37 | isopulegole acetate | -7.1 | Selina-6-en-4-ol | -6.9 |
| 38 | Aromadendrene | -7 | Trans-beta-santolol | -6.9 |
| 39 | Beta-bisabolene | -7 | Nerolidol | -6.8 |
| 40 | Cyclohexane,(2-nitro-2-propenyl) | -7 | 1-(beta.-d-Ribofuranosyl)-4-difluoromethyl-5-bromouracil | -6.8 |
| 41 | menth-3-en-9-ol | -7 | 3,7,11-tridecatrienoic-acid-4-8-12-trimethyl- | -6.8 |
| 42 | Nerylacetate | -7 | 3-methylbut-2-enoic acid,4-nitrophenyl ester | -6.8 |
| 43 | P-menth-1,8-dien-10-al | -7 | 5-epi-neointermedeol | -6.8 |
| 44 | Squalene | -7 | Alpha-cadinene | -6.8 |
| 45 | 8-hydroxymenthone | -6.9 | Alpha-cadinol | -6.8 |
| 46 | 1-(Hydroxymethyl)-4-(4-metho-xyphenyl)-10-oxa-4-aza-tricyclo-[5-2-1-0-20-6]-dec-8-ene-3,_5-dione | -6.9 | Epi-α-cadinol_ | -6.8 |
| 47 | 1-propanone-2-chloro-1-(2-5-dimethylphenyl)-2-methyl | -6.9 | Fenchyl_acetate | -6.8 |
| 48 | 4-hydroxylisopulegone | -6.9 | Gamma-cadinene | -6.8 |
| 49 | 8-acetoxylmenthone | -6.9 | hexamethyl-bicyclopentyl-2- | -6.8 |
| 50 | Azulene | -6.9 | Isospathulenol | -6.8 |
| 51 | Cis-p-menth-2-en-1-ol | -6.9 | Ledene-oxide-II | -6.8 |
| 52 | Cis-p-mentha-1,8-dien-2-ol | -6.9 | Trans-cadina-1(2),4-diene | -6.8 |
| 53 | Cuminaldehyde | -6.9 | Trans-nerolidol | -6.8 |
| 54 | Epi-α-cadinol_ | -6.9 | Viridiflorol | -6.8 |
| 55 | Farnesol | -6.9 | 2-(trifluoromethyl)benzothiazole | -6.7 |
| 56 | Isospathulenol | -6.9 | Alpha calacorene | -6.7 |
| 57 | Napthalene | -6.9 | Alpha-gurjunene | -6.7 |
| 58 | Nerylpropanoatr | -6.9 | Alpha-Selinene | -6.7 |
| 59 | P-menth-1,8-dien-10-ol | -6.9 | Beta-damascenone | -6.7 |
| 60 | 2-(trifluoromethyl)benzothiazole | -6.8 | Beta-selinene | -6.7 |
| 61 | Allo-Aromadendrene | -6.8 | Beta-vatirenene | -6.7 |
| 62 | Alpha-farnesene | -6.8 | Cadin-4-en-10-ol | -6.7 |
| 63 | Aristolone | -6.8 | Cedrelanol | -6.7 |
| 64 | Benzenepropanol-2-4-6-trimethyl- | -6.8 | Cyclohexadecane | -6.7 |
| 65 | Beta-cubebene | -6.8 | Epi-cubebol | -6.7 |
| 66 | Buchu camphor | -6.8 | Eudesma-4(15),7-dien-3-b-ol | -6.7 |
| 67 | Caryophyllene | -6.8 | Eudesmol | -6.7 |
| 68 | Cis-acetoxy pulegone | -6.8 | Gamma-muurolene | -6.7 |
| 69 | Cumin alcohol | -6.8 | Intermedeol | -6.7 |
| 70 | Diosphenol | -6.8 | Pinol | -6.7 |
| 71 | Epiglobulol | -6.8 | 4-epi-cubedol | -6.6 |
| 72 | Eudesmol | -6.8 | Alpha-amorphene | -6.6 |
| 73 | Gamma-selinene | -6.8 | Aristolone | -6.6 |
| 74 | Lavandulyl acetate | -6.8 | Borneol | -6.6 |
| 75 | Ledene-oxide-II | -6.8 | Caryophylla-2(12),6(13)-dien-5-one | -6.6 |
| 76 | P-Menth-1-en-9-al | -6.8 | Cubebol | -6.6 |
| 77 | P-mentth-1,4-dien-7-ol | -6.8 | Elemol | -6.6 |
| 78 | Spathulenol | -6.8 | Germacrene-D-4-ol | -6.6 |
| 79 | 2-octanoylfuran | -6.7 | m-Toluamide | -6.6 |
| 80 | Alpha-bisabolo | -6.7 | Myrtanol | -6.6 |
| 81 | Alpha-terpinene | -6.7 | Myrtanol acetate | -6.6 |
| 82 | Alpha-thujenal | -6.7 | Myrtenol | -6.6 |
| 83 | Beta-bourbonene | -6.7 | Shyobunone | -6.6 |
| 84 | Beta-selinene | -6.7 | Trans-bergamotene | -6.6 |
| 85 | Carvacrol | -6.7 | Trifluoroacetyllavandulol | -6.6 |
| 86 | Cubenol | -6.7 | 1,8-Cineol | -6.5 |
| 87 | Dihdrocarvone | -6.7 | 1,4-Cineol | -6.5 |
| 88 | Dodeca-cis-cis-3,6-dienylacetate | -6.7 | 3-methylbut-2-enoic acid,3,4-dinitrophenyl ester | -6.5 |
| 89 | Gamma-Terpinene | -6.7 | 8-acetylthio-p-menthan-3-one | -6.5 |
| 90 | Geranylacetate | -6.7 | Alpha-ylangene | -6.5 |
| 91 | Jasmone | -6.7 | Alpha-pinene | -6.5 |
| 92 | menthofulran | -6.7 | Beta- costol | -6.5 |
| 93 | Oxalic acid-2-methylphenylpentadecylester | -6.7 | Beta-bisabolene | -6.5 |
| 94 | P-cymene | -6.7 | Beta-caryophylleneoxide | -6.5 |
| 95 | Phenol-2-(4-diethylaminophenyl-iminomethyl)-_ | -6.7 | Camphene | -6.5 |
| 96 | P-menth-8-en-7-ol | -6.7 | Caryophyllene | -6.5 |
| 97 | Rosefuran-epoxide | -6.7 | Davanone | -6.5 |
| 98 | Tau-muurolol | -6.7 | Epi-a-muurolol | -6.5 |
| 99 | Nerolidol | -6.6 | Farnesol | -6.5 |
| 100 | 1,3,8-p-menthatriene | -6.6 | Octen-2-yn-4-ol | -6.5 |
| 101 | 3,7,11-tridecatrienoic-acid-4-8-12-trimethyl- | -6.6 | Spathulenol | -6.5 |
| 102 | 3-methyl-2-butenoic acid,oct-3-en-2-yl ester | -6.6 | Trans(beta)-caryuophyllene oxide | -6.5 |
| 103 | 3-p-menthene | -6.6 | Isomenthone | -6.4 |
| 104 | 4-acetoxylisopulegone | -6.6 | 3-hexenyl-benzoate | -6.4 |
| 105 | Aceticacid-1,3,7-trimethylocta-2-6-dienyl | -6.6 | 3-methylbut-2-enoic acid,2,3,4,6-tetrachlorophenyl ester | -6.4 |
| 106 | Alpha-copaene | -6.6 | 3-methylbut-2-enoic acid,2-methyl oct-5-yn-4-yl ester | -6.4 |
| 107 | 8,8'-Bi-p-cymene | -6.6 | 4-acetoxylisopulegone | -6.4 |
| 108 | Benzyl-benzoate | -6.6 | 4-Isopropyl-6-methyltetralone | -6.4 |
| 109 | Cymopyrocatechol | -6.6 | 9,10-dehydroisolongifolene | -6.4 |
| 110 | Davanone | -6.6 | Alpha-copaene | -6.4 |
| 111 | Eicosatrenoic acid | -6.6 | Beta-cubebene | -6.4 |
| 112 | Isoeugenol | -6.6 | Beta-ionone | -6.4 |
| 113 | M-cymene | -6.6 | caryophylla-3(15),7(14)-dien-6-ol | -6.4 |
| 114 | Neric-acid | -6.6 | Caryophylla-4(14)-8(15)-dien-5-ol | -6.4 |
| 115 | P-cymen-8-ol | -6.6 | Cis-acetoxy pulegone | -6.4 |
| 116 | Piperitone | -6.6 | D-elemene | -6.4 |
| 117 | P-menth-4(8)-en-7-ol | -6.6 | Eicosatrenoic acid | -6.4 |
| 118 | P-menth-1,3,6-triene | -6.6 | Epiglobulol | -6.4 |
| 119 | Pulegone | -6.6 | Isocaryophyllene | -6.4 |
| 120 | Trans-nerolidol | -6.6 | isopulegole acetate | -6.4 |
| 121 | 3-thujanol | -6.5 | Trans-alpha-bergamotol | -6.4 |
| 122 | Alpha-ylangene | -6.5 | 1-(-4-methoxylphenyl)-1H-pyrrolle-2-5-dione | -6.3 |
| 123 | Alpha-phellandrene | -6.5 | 1H-Benzimidazol-2-amine | -6.3 |
| 124 | Alpha-Selinene | -6.5 | 2-Hexadecen-1-ol-3,7,11,15-tetramethyl | -6.3 |
| 125 | Artemisia acetate | -6.5 | 3-(Octadecyloxy)-propyl_ester | -6.3 |
| 126 | Beta-farnesene | -6.5 | 4-Acetoxylisomenthone | -6.3 |
| 127 | Beta-ionone | -6.5 | 6,10-Dimethylundeca-1-5-9-trien-4-ol | -6.3 |
| 128 | Beta-vatirenene | -6.5 | 8-acetoxylmenthone | -6.3 |
| 129 | Carvone | -6.5 | Aceticacid-1,3,7-trimethylocta-2-6-dienyl | -6.3 |
| 130 | Cis-carveol | -6.5 | Allo-Aromadendrene | -6.3 |
| 131 | Delta-terpineol | -6.5 | Alpha-bergamotene | -6.3 |
| 132 | Durene | -6.5 | Beta-bourbonene | -6.3 |
| 133 | Durenol | -6.5 | Buchu camphor | -6.3 |
| 134 | isopulegone | -6.5 | Cedren-13-ol | -6.3 |
| 135 | Limonene | -6.5 | Cis-carvylacetate | -6.3 |
| 136 | m-Toluamide | -6.5 | Cis-elema-1-3-dien-7-ol | -6.3 |
| 137 | Nerylformate | -6.5 | Diosphenol | -6.3 |
| 138 | P-menth-1(7)-en-9-ol | -6.5 | Geranylacetate | -6.3 |
| 139 | Spiro-[-4-5-]-decan-2-one | -6.5 | Humulene | -6.3 |
| 140 | Terpinolene | -6.5 | L-Menthyl_acetate | -6.3 |
| 141 | Trans-alpha-bergamotol | -6.5 | P-menth-1-en-9-yl-acetate | -6.3 |
| 142 | Trans-Isopulegone | -6.5 | Trans-carveyl acetate | -6.3 |
| 143 | Trans-piperitol | -6.5 | 3-methyl-2-butenoic acid,oct-3-en-2-yl ester | -6.2 |
| 144 | Trans-sabinol | -6.5 | Alpha-muurolol | -6.2 |
| 145 | 3-methylbut-2-enoic acid,2-methyl oct-5-yn-4-yl ester | -6.4 | Alpha-phellandrene | -6.2 |
| 146 | 3-thujanone | -6.4 | Beta-copaene | -6.2 |
| 147 | 5-epi-neointermedeol | -6.4 | Beta-gurjunene | -6.2 |
| 148 | Alpha-bergamotene | -6.4 | Carvacrol | -6.2 |
| 149 | Alpha-terpineol | -6.4 | Cymopyrocatechol | -6.2 |
| 150 | Alpha-thujone | -6.4 | Dodeca-cis-cis-3,6-dienylacetate | -6.2 |
| 151 | Beta-phellandrene | -6.4 | Isoeugenol | -6.2 |
| 152 | Cedren-13-ol | -6.4 | Piperitone | -6.2 |
| 153 | Cis-4-(isopropyl)-1-methylcyclohex-2-en-1-ol | -6.4 | Pulegone | -6.2 |
| 154 | Cis-limonene oxide | -6.4 | Trans-2-acetoxylpulegone | -6.2 |
| 155 | Cis-piperitol | -6.4 | Trans-2-hydroxylmenthone | -6.2 |
| 156 | Citral | -6.4 | Trans-sabinene-hydrate | -6.2 |
| 157 | Diosphenol-methyl ether | -6.4 | 6,10,14-trimethyl-2-pentadecanone | -6.1 |
| 158 | D-Limonene | -6.4 | Benzenepropanol-2-4-6-trimethyl- | -6.1 |
| 159 | Gamma-terpineol | -6.4 | Bicyclogermacrene | -6.1 |
| 160 | Geranyl-n-butyrate | -6.4 | Cis-trans-4-n-propyl-3-oxabicyclo-[4.4.0]decane | -6.1 |
| 161 | Germacrene_A | -6.4 | Humulene-epoxide-II | -6.1 |
| 162 | Humulene | -6.4 | Linalool acetate | -6.1 |
| 163 | Menthol | -6.4 | Methyllinolenate | -6.1 |
| 164 | Nerolacetate | -6.4 | Methyl-10,12-pentacosadiynoate | -6.1 |
| 165 | Tetrahydrolavandulylacetate | -6.4 | P-cymene | -6.1 |
| 166 | Trans-2-acetoxylpulegone | -6.4 | Phytol | -6.1 |
| 167 | Trans-carveol | -6.4 | P-menth-4(8)-en-7-ol | -6.1 |
| 168 | 3-hexenyl-benzoate | -6.3 | Trans-2-caren-4-ol | -6.1 |
| 169 | 3-hexenyl-isobutyrate | -6.3 | Trans-sabinol | -6.1 |
| 170 | 1-(-4-methoxylphenyl)-1H-pyrrolle-2-5-dione | -6.3 | 1,3,8-p-menthatriene | -6 |
| 171 | 2-Hexadecen-1-ol-3,7,11,15-tetramethyl | -6.3 | 1-propanone-2-chloro-1-(2-5-dimethylphenyl)-2-methyl | -6 |
| 172 | 6,10-Dimethylundeca-1-5-9-trien-4-ol | -6.3 | 3-methyl-2-butenoic acid,pent-2-en-4-yinyl ester | -6 |
| 173 | Alpha-thujene | -6.3 | 3-thujanol | -6 |
| 174 | Beta-damascenone | -6.3 | 4-Terpineol | -6 |
| 175 | D-elemene | -6.3 | 6-epi-shyobunol | -6 |
| 176 | Elemol | -6.3 | Berbenone | -6 |
| 177 | Eugenol | -6.3 | Beta-phellandrene | -6 |
| 178 | Germacrene_D | -6.3 | Bornyl acetate | -6 |
| 179 | Linoleic acid | -6.3 | Cis-verbenone | -6 |
| 180 | Methyleugenol | -6.3 | Cumin alcohol | -6 |
| 181 | neral | -6.3 | Linoleic acid | -6 |
| 182 | Sabinaketone | -6.3 | M-cymene | -6 |
| 183 | Sabinyl acetate | -6.3 | Methyleugenol | -6 |
| 184 | Trans-bergamotene | -6.3 | Neric-acid | -6 |
| 185 | Trans-beta-santolol | -6.3 | Nerylformate | -6 |
| 186 | Trans-p-meth-2-en-1-ol | -6.3 | Oxalic acid-2-methylphenylpentadecylester | -6 |
| 187 | 3-methylbut-2-enoic acid,2,3,4,6-tetrachlorophenyl ester | -6.2 | P-menth-1,3,6-triene | -6 |
| 188 | 3-methylbut-2-enoic acid,3,4-dinitrophenyl ester | -6.2 | Pseudodiosphenol | -6 |
| 189 | 4-thajunol | -6.2 | Pulegone-epoxide A | -6 |
| 190 | Beta- costol | -6.2 | Sabinyl acetate | -6 |
| 191 | Beta-gurjunene | -6.2 | Terpinene-4-ol | -6 |
| 192 | caryophylla-3(15),7(14)-dien-6-ol | -6.2 | Terpinolene | -6 |
| 193 | Caryophylla-4(14)-8(15)-dien-5-ol | -6.2 | 8-hydroxymenthone | -5.9 |
| 194 | Cis-sabinenehydrate | -6.2 | 3-hexenyl-2-methylbutyrate | -5.9 |
| 195 | Cubebol | -6.2 | Alpha-thujene | -5.9 |
| 196 | Geraniol | -6.2 | Beta-ocimene | -5.9 |
| 197 | Geranyl-formate | -6.2 | Carvone | -5.9 |
| 198 | Germacrene-D-4-ol | -6.2 | Chrysanthenone | -5.9 |
| 199 | hexamethyl-bicyclopentyl-2- | -6.2 | Cis-1,2-epoxy-terpin-4-ol | -5.9 |
| 200 | Hexanoic acid | -6.2 | Cis-chryanthenol | -5.9 |
| 201 | p-isopropylphenol | -6.2 | Cis-chrysantheneyl acetate | -5.9 |
| 202 | Trans-sabinene-hydrate | -6.2 | Cis-limonene oxide | -5.9 |
| 203 | 3-hexenyl-2-methylbutyrate | -6.1 | Cis-p-mentha-1,8-dien-2-ol | -5.9 |
| 204 | 4-Terpineol | -6.1 | Cis-verbenol | -5.9 |
| 205 | Beta-ocimene | -6.1 | Cuminaldehyde | -5.9 |
| 206 | Citronellal | -6.1 | Diosphenol-methyl ether | -5.9 |
| 207 | Dehydrosabinaketone | -6.1 | D-Limonene | -5.9 |
| 208 | Estragole | -6.1 | Gamma-terpineol | -5.9 |
| 209 | Ethanone-1-(1-4-dimethyl-3-cyclohexen-1-yl)- | -6.1 | Heneicosane | -5.9 |
| 210 | Globulol | -6.1 | isoneomenthol | -5.9 |
| 211 | Nerol | -6.1 | isopulegone | -5.9 |
| 212 | Pentylbenzene | -6.1 | Methyl-elaidate | -5.9 |
| 213 | Phytol | -6.1 | Neomenthol | -5.9 |
| 214 | Rose-oxide | -6.1 | neral | -5.9 |
| 215 | Sabinene | -6.1 | Nerol | -5.9 |
| 216 | Terpinene-4-ol | -6.1 | Nerolacetate | -5.9 |
| 217 | Trans-chrysanthemal | -6.1 | Nerylacetate | -5.9 |
| 218 | Trans-p-menth-2,8-dien-1-ol | -6.1 | P-cymen-8-ol | -5.9 |
| 219 | Ursolic_acid (reference compound) | -6.1 | P-menth-1,8-dien-10-al | -5.9 |
| 220 | Isomenthone | -6 | P-mentth-1,4-dien-7-ol | -5.9 |
| 221 | 2-methylbutyl isovalerate | -6 | Rosefuran-epoxide | -5.9 |
| 222 | Acetophone | -6 | Trans-carveol | -5.9 |
| 223 | Alpha-amorphene | -6 | Trans-Isopulegone | -5.9 |
| 224 | Beta-linalool | -6 | Trans-piperitol | -5.9 |
| 225 | Citronellol | -6 | Trans-verbenol | -5.9 |
| 226 | Cyclopentanepropanol-2-methylene | -6 | 3-hexenyl-isobutyrate | -5.8 |
| 227 | Linalool acetate | -6 | 1-pyrazinyl-4-methyl-2-pentanol | -5.8 |
| 228 | Methyl-elaidate | -6 | 2,6-dimethyl-1-2-5-7-octatetraene-E-E- | -5.8 |
| 229 | Neoisopulegol | -6 | 2-hexanoic-acid-3,4,4-trimethyl-5-oxo-Z- | -5.8 |
| 230 | Pseudodiosphenol | -6 | 2-methylbenzoxazole | -5.8 |
| 231 | Trans-beta-ocimene | -6 | 2-Nonadeconone | -5.8 |
| 232 | 1,2,4,4-tetramethylcyclopentene | -5.9 | 3-thujanone | -5.8 |
| 233 | 1-pyrazinyl-4-methyl-2-pentanol | -5.9 | 4-hydroxylisopulegone | -5.8 |
| 234 | 2-benzyl-2-5-5-trimethyl-1-3-dioxane | -5.9 | Allo-ocimine | -5.8 |
| 235 | 2-hexanoic-acid-3,4,4-trimethyl-5-oxo-Z- | -5.9 | Alpha-terpineol | -5.8 |
| 236 | 3-methyl-2-butenoic acid,pent-2-en-4-yinyl ester | -5.9 | Azulene | -5.8 |
| 237 | 4-hydroxyl-4-methylcyclohex-2-en-1-one | -5.9 | Beta-linalool | -5.8 |
| 238 | Alpha-muurolene | -5.9 | Beta-myrcene | -5.8 |
| 239 | Artemisia ketone | -5.9 | Cis-carveol | -5.8 |
| 240 | Cis-beta-ocimene | -5.9 | Dihdrocarvone | -5.8 |
| 241 | Eudesma-4(15),7-dien-3-b-ol | -5.9 | Epoxylmycrene | -5.8 |
| 242 | Lavandulol | -5.9 | Estragole | -5.8 |
| 243 | Linalool | -5.9 | Eugenol | -5.8 |
| 244 | Myrtanol acetate | -5.9 | Gamma-Terpinene | -5.8 |
| 245 | 1-(beta.-d-Ribofuranosyl)-4-difluoromethyl-5-bromouracil | -5.8 | Isothujol | -5.8 |
| 246 | 2,6-dimethyl-1-2-5-7-octatetraene-E-E- | -5.8 | Linalool | -5.8 |
| 247 | 2-methylbenzoxazole | -5.8 | Linalool-oxide | -5.8 |
| 248 | 2-thujene | -5.8 | menthofulran | -5.8 |
| 249 | 6,10,14-trimethyl-2-pentadecanone | -5.8 | Menthol | -5.8 |
| 250 | Alpha-muurolol | -5.8 | Mycrene | -5.8 |
| 251 | Artemisia alcohol | -5.8 | Rose-oxide | -5.8 |
| 252 | Beta-caryophylleneoxide | -5.8 | Santolinyl acetate | -5.8 |
| 253 | Beta-copaene | -5.8 | Trans-linalool oxide | -5.8 |
| 254 | Beta-myrcene | -5.8 | Verbenol | -5.8 |
| 255 | Beta-thujene | -5.8 | 2-Cyclohexen-1-ol-2-methyl-5-(1-methyethenyl)-cis- | -5.7 |
| 256 | Bicyclogermacrene | -5.8 | 2-Tridecanone | -5.7 |
| 257 | Cis-trans-4-n-propyl-3-oxabicyclo-[4.4.0]decane | -5.8 | 3-p-menthene | -5.7 |
| 258 | Epoxylmycrene | -5.8 | 6-Methyloctadecane | -5.7 |
| 259 | Isothujol | -5.8 | Alphacyclogeraniolacetate | -5.7 |
| 260 | Myrtenyl acetate | -5.8 | Alpha-terpinene | -5.7 |
| 261 | phenethylalcohol | -5.8 | Bicycloelemene | -5.7 |
| 262 | 1H-Benzimidazol-2-amine | -5.7 | D-3-carene | -5.7 |
| 263 | 2-Tridecanone | -5.7 | Delta-terpineol | -5.7 |
| 264 | 2-undecanone | -5.7 | Durenol | -5.7 |
| 265 | 3-methyl-2-butenoic acid,cyclobutyl ester | -5.7 | Ethanone-1-(1-4-dimethyl-3-cyclohexen-1-yl)- | -5.7 |
| 266 | Allo-ocimine | -5.7 | Fenchone | -5.7 |
| 267 | Alpha-ocimene | -5.7 | Geraniol | -5.7 |
| 268 | Caryophylla-2(12),6(13)-dien-5-one | -5.7 | Geranyl-formate | -5.7 |
| 269 | Epi-cyclogeraniolene | -5.7 | Hepadecane | -5.7 |
| 270 | Hydroxylcitronellal | -5.7 | Jasmone | -5.7 |
| 271 | Mycrene | -5.7 | Lavandulyl acetate | -5.7 |
| 272 | Selina-6-en-4-ol | -5.7 | Limonene | -5.7 |
| 273 | Shyobunone | -5.7 | Menthone | -5.7 |
| 274 | 2-carene | -5.6 | Methylstearate | -5.7 |
| 275 | 2-dodecanone | -5.6 | Napthalene | -5.7 |
| 276 | Alpha-acorenol | -5.6 | Neoisopulegol | -5.7 |
| 277 | Beta-pinene | -5.6 | Nerylpropanoatr | -5.7 |
| 278 | Citronellene | -5.6 | Nonane | -5.7 |
| 279 | isoneomenthol | -5.6 | p-isopropylphenol | -5.7 |
| 280 | Methylstearate | -5.6 | P-menth-8-en-7-ol | -5.7 |
| 281 | Neomenthol | -5.6 | Trans-8-mercapto-p-mentha-3-one | -5.7 |
| 282 | Santolina alcohol | -5.6 | Trans-pinocamphone | -5.7 |
| 283 | Seudenone | -5.6 | Trans-p-menth-2,8-dien-1-ol | -5.7 |
| 284 | 2-(1-cyano-2-methyl-propylamino)-3-methyl | -5.5 | 2-octanoylfuran | -5.6 |
| 285 | 2-Acetylfuran | -5.5 | 2-thujene | -5.6 |
| 286 | 2-Nonadeconone | -5.5 | 4-thajunol | -5.6 |
| 287 | 3-methylcyclohexanone | -5.5 | Alpha-thujenal | -5.6 |
| 288 | Alphacyclogeraniolacetate | -5.5 | Artemisia acetate | -5.6 |
| 289 | Cis-dec-4-enol | -5.5 | Artemisia ketone | -5.6 |
| 290 | Cis-verbenol | -5.5 | Cis-4-(isopropyl)-1-methylcyclohex-2-en-1-ol | -5.6 |
| 291 | Hexahydropentalene | -5.5 | Citral | -5.6 |
| 292 | Isoamyl isovalerate | -5.5 | Methyl-palmitate | -5.6 |
| 293 | Methylheptanone | -5.5 | Pentylbenzene | -5.6 |
| 294 | Pemtanoic acid, 2-methylbutyl ester | -5.5 | P-Menth-1-en-9-al | -5.6 |
| 295 | Trans(beta)-caryuophyllene oxide | -5.5 | P-menth-1,8-dien-10-ol | -5.6 |
| 296 | Trans-8-mercapto-p-mentha-3-one | -5.5 | Trans-beta-ocimene | -5.6 |
| 297 | Verbenol | -5.5 | Trans-carene-4,5-epoxide | -5.6 |
| 298 | Yomgi Alcohol | -5.5 | Trans-chrysanthemal | -5.6 |
| 299 | 2-Cyclohexen-1-ol-2-methyl-5-(1-methyethenyl)-cis- | -5.4 | Trans-pinocarveol | -5.6 |
| 300 | 2-methyl-5-(1-propenyl)pyrazine | -5.4 | Trans-p-meth-2-en-1-ol | -5.6 |
| 301 | 4-Acetoxylisomenthone | -5.4 | Triacontane | -5.6 |
| 302 | 8-acetylthio-p-menthan-3-one | -5.4 | 2-carene | -5.5 |
| 303 | D-3-carene | -5.4 | 2-dodecanone | -5.5 |
| 304 | Decanal | -5.4 | 2-methyl-5-(1-propenyl)pyrazine | -5.5 |
| 305 | Fenchone | -5.4 | Acetophone | -5.5 |
| 306 | Gitoxigenin | -5.4 | Artemisia alcohol | -5.5 |
| 307 | Intermedeol | -5.4 | Beta-thujene | -5.5 |
| 308 | Linalool-oxide | -5.4 | Cis-beta-ocimene | -5.5 |
| 309 | Methyl-palmitate | -5.4 | Cis-piperitol | -5.5 |
| 310 | Nonane | -5.4 | Cis-p-menth-2-en-1-ol | -5.5 |
| 311 | octadecanol | -5.4 | Citronellene | -5.5 |
| 312 | Octen-2-yn-4-ol | -5.4 | Hexanoic acid | -5.5 |
| 313 | Pinol | -5.4 | Nonacosane | -5.5 |
| 314 | Pulegone-epoxide A | -5.4 | Octacosane | -5.5 |
| 315 | Santolinatriene | -5.4 | Pemtanoic acid, 2-methylbutyl ester | -5.5 |
| 316 | Trans-carene-4,5-epoxide | -5.4 | P-menth-1(7)-en-9-ol | -5.5 |
| 317 | Trans-dec-4-enol | -5.4 | Spiro-[-4-5-]-decan-2-one | -5.5 |
| 318 | Trans-linalool oxide | -5.4 | Tetrahydrolavandulylacetate | -5.5 |
| 319 | Vinycyclohexane | -5.4 | Tricosane | -5.5 |
| 320 | Viridiflorol | -5.4 | 2-methylbutyl isovalerate | -5.4 |
| 321 | 6-epi-shyobunol | -5.3 | 2-undecanone | -5.4 |
| 322 | Alpha-gurjunene | -5.3 | Alpha-thujone | -5.4 |
| 323 | Cis-1,2-epoxy-terpin-4-ol | -5.3 | Beta-pinene | -5.4 |
| 324 | Cis-elema-1-3-dien-7-ol | -5.3 | Citronellol | -5.4 |
| 325 | Guaiacol | -5.3 | Cyclohexane,(2-nitro-2-propenyl) | -5.4 |
| 326 | Menthone | -5.3 | Dehydrosabinaketone | -5.4 |
| 327 | Methyl-10,12-pentacosadiynoate | -5.3 | Epi-cyclogeraniolene | -5.4 |
| 328 | Nonadecane | -5.3 | Hydroxylcitronellal | -5.4 |
| 329 | Trans-2-caren-4-ol | -5.3 | Isoamyl isovalerate | -5.4 |
| 330 | 2-octanone | -5.2 | menth-3-en-9-ol | -5.4 |
| 331 | 3-(Octadecyloxy)-propyl_ester | -5.2 | Octadecane | -5.4 |
| 332 | 6-Methyloctadecane | -5.2 | phenethylalcohol | -5.4 |
| 333 | Camphene | -5.2 | Sabinene | -5.4 |
| 334 | Heneicosane | -5.2 | 2-(1-cyano-2-methyl-propylamino)-3-methyl | -5.3 |
| 335 | Hepadecane | -5.2 | 3-methyl-2-butenoic acid,cyclobutyl ester | -5.3 |
| 336 | Methyllinolenate | -5.2 | Cis-sabinenehydrate | -5.3 |
| 337 | Nonanal | -5.2 | Citronellal | -5.3 |
| 338 | Octadecane | -5.2 | Dehydro-1,8-cineole | -5.3 |
| 339 | 1-octen-3-ol | -5.1 | Durene | -5.3 |
| 340 | 5-methylfufural | -5.1 | Eucalyptol | -5.3 |
| 341 | Chrysanthenone | -5.1 | Lavandulol | -5.3 |
| 342 | Humulene-epoxide-II | -5.1 | octadecanol | -5.3 |
| 343 | Octacosane | -5.1 | Sabinaketone | -5.3 |
| 344 | Santolinyl acetate | -5.1 | Santolina alcohol | -5.3 |
| 345 | Tricyclene | -5.1 | Tricyclene | -5.3 |
| 346 | 1,4-Cineol | -5 | Vinycyclohexane | -5.3 |
| 347 | 9,10-dehydroisolongifolene | -5 | 3-methylcyclohexanone | -5.2 |
| 348 | Furfural | -5 | Alpha-Fenchene | -5.2 |
| 349 | Myrtanol | -5 | Guaiacol | -5.2 |
| 350 | Nonacosane | -5 | Seudenone | -5.2 |
| 351 | Tricosane | -5 | Yomgi Alcohol | -5.2 |
| 352 | 1,8-Cineol | -4.9 | 2,7-octadiene-4-methyl | -5.1 |
| 353 | 2,7-octadiene-4-methyl | -4.9 | 4-hydroxyl-4-methylcyclohex-2-en-1-one | -5.1 |
| 354 | Alpha-Fenchene | -4.9 | 5-methylfufural | -5.1 |
| 355 | Bicycloelemene | -4.9 | Alpha-ocimene | -5.1 |
| 356 | Bornyl acetate | -4.9 | Cis-dec-4-enol | -5.1 |
| 357 | Cis-chrysantheneyl acetate | -4.9 | Cyclopentanepropanol-2-methylene | -5.1 |
| 358 | Myrtenol | -4.9 | Methylheptanone | -5.1 |
| 359 | Pinacol | -4.9 | Santolinatriene | -5.1 |
| 360 | N-chlorosuccinimide | -4.8 | Tetracontane | -5.1 |
| 361 | Triacontane | -4.8 | Trans-dec-4-enol | -5.1 |
| 362 | Cis-verbenone | -4.7 | 1,2,4,4-tetramethylcyclopentene | -5 |
| 363 | Fenchyl_acetate | -4.7 | 2-benzyl-2-5-5-trimethyl-1-3-dioxane | -5 |
| 364 | Pirilene | -4.7 | Decanal | -5 |
| 365 | pyrazinamide | -4.7 | Hexahydropentalene | -5 |
| 366 | Trans-verbenol | -4.7 | Pirilene | -5 |
| 367 | Alpha-pinene | -4.6 | pyrazinamide | -5 |
| 368 | Berbenone | -4.6 | 2-Acetylfuran | -4.9 |
| 369 | Dehydro-1,8-cineole | -4.6 | Nonanal | -4.9 |
| 370 | Trans-pinocamphone | -4.6 | 1-octen-3-ol | -4.8 |
| 371 | Trans-pinocarveol | -4.6 | Nonadecane | -4.8 |
| 372 | Cis-chryanthenol | -4.5 | Gitoxigenin | -4.7 |
| 373 | Eucalyptol | -4.5 | 2-octanone | -4.6 |
| 374 | Tetracontane | -4.5 | N-chlorosuccinimide | -4.6 |
| 375 | 1,3-dibromopentane | -4.1 | Pinacol | -4.5 |
| 376 | Borneol | -4.1 | Histamine | -4.4 |
| 377 | 1,3-cyclopentadiene | -4 | Furfural | -4.3 |
| 378 | Histamine | -4 | 1,3-dibromopentane | -4.1 |
| 379 | Trans-2-hydroxylmenthone | -4 | 1,3-cyclopentadiene | -3.7 |

Table S2. Correlation analysis between binding energy and other thermodynamic parameters

|  | *ΔΕvdW* | *ΔEelec* | *ΔEGB* | *ΔEsurf* | *ΔGgas* | *Δsolv* | *ΔGbind* |
| --- | --- | --- | --- | --- | --- | --- | --- |
| ΔΕvdW | 1 |  |  |  |  |  |  |
| ΔEelec | 0.288706 | 1 |  |  |  |  |  |
| ΔEGB | 0.26319 | 0.997253 | 1 |  |  |  |  |
| ΔEsurf | 0.326684 | 0.104767 | 0.072789 | 1 |  |  |  |
| ΔGgas | 0.397329 | 0.993309 | 0.987534 | 0.139493 | 1 |  |  |
| Δsolv | 0.243945 | 0.99625 | 0.999792 | 0.065928 | 0.984249 | 1 |  |
| ΔGbind | 0.924142 | 0.323576 | 0.274095 | 0.427013 | 0.421946 | 0.255031 | 1 |
